# In-depth phenotyping for clinical stratification of Gaucher disease

**DOI:** 10.1186/s13023-021-02034-6

**Published:** 2021-10-14

**Authors:** Simona D’Amore, Kathleen Page, Aimée Donald, Khadijeh Taiyari, Brian Tom, Patrick Deegan, Chong Y. Tan, Kenneth Poole, Simon A. Jones, Atul Mehta, Derralynn Hughes, Reena Sharma, Robin H. Lachmann, Anupam Chakrapani, Tarekegn Geberhiwot, Saikat Santra, Siddarth Banka, Timothy M. Cox, the MRC GAUCHERITE Consortium, T. M. Cox, F. M. Platt, S. Banka, A. Chakrapani, P. B. Deegan, T. Geberhiwot, D. A. Hughes, S. Jones, R. H. Lachmann, S. Santra, R. Sharma, A. Vellodi

**Affiliations:** 1 Department of Medicine, University of Cambridge, Cambridge, UK; 2 Medical Research Council Biostatistics Unit, University of Cambridge, Cambridge, UK; 3 Cambridge University Hospitals, Cambridge, UK; 4 Manchester Centre for Genomic Medicine, St Mary’s Hospital, Manchester University NHS Foundation Trust, University of Manchester, Manchester, UK; 5 Royal Free Hospital, London, UK; 6 Salford Royal NHS Foundation Trust, Salford, UK; 7 National Hospital for Neurology and Neurosurgery, London, UK; 8 Great Ormond Street Hospital, London, UK; 9 Birmingham Queen Elizabeth Hospital, Birmingham, UK; 10 Birmingham Children’s Hospital, Birmingham, UK; 11 Present Address: Manchester Centre for Genomic Medicine, St Mary’s Hospital, Manchester, UK; 12 Present Address: Centre for Trials Research, Cardiff University, Cardiff, UK

**Keywords:** Gaucher disease, Cohort, GAUCHERITE, Enzyme replacement therapy, Substrate reduction therapy, Disease-modifying therapies

## Abstract

**Background:**

The Gaucher Investigative Therapy Evaluation is a national clinical cohort of 250 patients aged 5–87 years with Gaucher disease in the United Kingdom—an ultra-rare genetic disorder. To inform clinical decision-making and improve pathophysiological understanding, we characterized the course of Gaucher disease and explored the influence of costly innovative medication and other interventions. Retrospective and prospective clinical, laboratory and radiological information including molecular analysis of the *GBA*1 gene and comprising > 2500 variables were collected systematically into a relational database with banking of collated biological samples in a central bioresource. Data for deep phenotyping and life-quality evaluation, including skeletal, visceral, haematological and neurological manifestations were recorded for a median of 17.3 years; the skeletal and neurological manifestations are the main focus of this study.

**Results:**

At baseline, 223 of the 250 patients were classified as type 1 Gaucher disease. Skeletal manifestations occurred in most patients in the cohort (131 of 201 specifically reported bone pain). Symptomatic osteonecrosis and fragility fractures occurred respectively in 76 and 37 of all 250 patients and the first osseous events occurred significantly earlier in those with neuronopathic disease. Intensive phenotyping in a subgroup of 40 patients originally considered to have only systemic features, revealed neurological involvement in 18: two had Parkinson disease and 16 had clinical signs compatible with neuronopathic Gaucher disease—indicating a greater than expected prevalence of neurological features. Analysis of longitudinal real-world data enabled Gaucher disease to be stratified with respect to advanced therapies and splenectomy. Splenectomy was associated with an increased hazard of fragility fractures, in addition to osteonecrosis and orthopaedic surgery; there were marked gender differences in fracture risk over time since splenectomy. Skeletal disease was a heavy burden of illness, especially where access to specific therapy was delayed and in patients requiring orthopaedic surgery.

**Conclusion:**

Gaucher disease has been explored using real-world data obtained in an era of therapeutic transformation. Introduction of advanced therapies and repeated longitudinal measures enabled this heterogeneous condition to be stratified into obvious clinical endotypes. The study reveals diverse and changing phenotypic manifestations with systemic, skeletal and neurological disease as inter-related sources of disability.

**Supplementary Information:**

The online version contains supplementary material available at 10.1186/s13023-021-02034-6.

## Background

Gaucher disease is an autosomal recessive disorder of sphingolipid metabolism caused by catalytic deficiency of lysosomal acid β-D-glucosylceramidase (ß-glucocerebrosidase; EC 3.2.1.45) [1, 2]. Impaired lysosomal recycling of ß-glucosylceramides leads to over-production of ß-glucosylsphingosine. This ultra-rare inborn error is principally caused by biallelic mutations in the *GBA*1 gene located on chromosome 1q22 [2] and is most frequent in Ashkenazi Jews and occurs in small isolated populations in North Sweden and Brazil [3–5].

A convenient, but imperfect clinical classification of Gaucher disease, recognises three principal subtypes: type 1 (GD1, OMIM 230800, chronic non-neuronopathic), type 2 (GD2, OMIM 230900, acute neuronopathic) and type 3 (GD3, OMIM 231000, chronic neuronopathic) diseases [2]. This operational classification is summarized in Additional file 1: Table S1. However, a striking feature in all subtypes, is the heterogeneity of this disease in the age and rate of onset, as well as the widespread effects. Especially in the type 1 disease, adults as well as children can have only mild symptoms or be asymptomatic. Marked differences of disease expression are seen between affected siblings and clinically discordant monozygotic twins suggest not only the existence of modifier genes but complex interactions with environmental factors [2, 6–9].

**Fig. 1:**
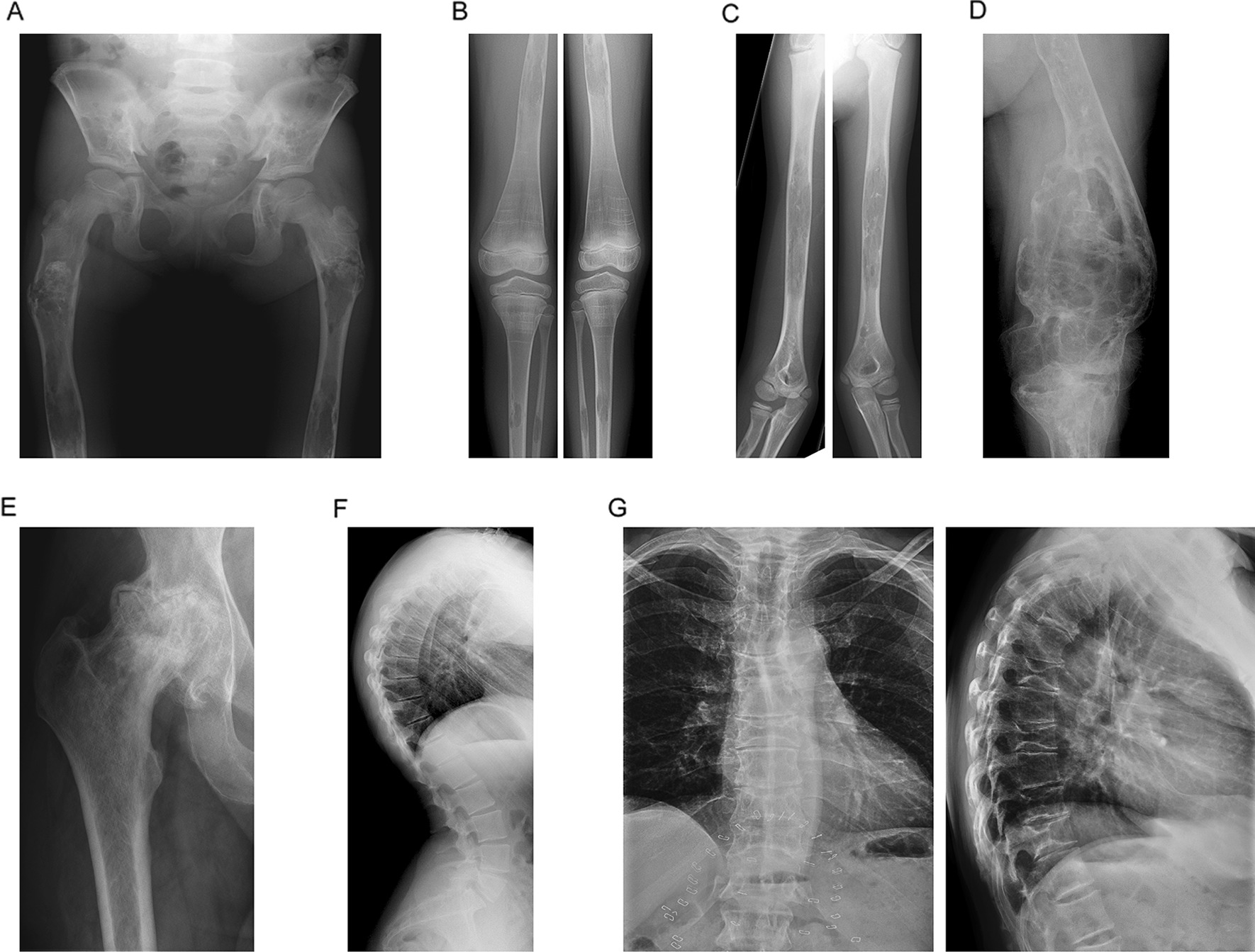
Radiographic imaging of skeletal manifestations in Gaucher disease. Generalized osteopaenia and lytic areas in iliac wings (**A**) and mid shafts of both femora (**A**, **B**), tibiae (**B**) and humeri (**C**). Pathological fractures both upper femora (**A**). Erlenmeyer flask deformity of both femurs, also known as metaphyseal flaring (**B**). Gross expansion and coarse trabeculation of the left femur (**D**). Extensive osteonecrosis of the right femoral head, which shows marked flattening with complete loss of joint space (**E**). Prominent thoracic kyphosis and exaggerated lumbar lordosis with minimal lumbar curve (**F**). H-shaped vertebrae of the thoracic spine consistent with osteonecrosis (**G**)

Evidence of disease is detectable in the bone marrow and macrophage-rich organs such as the spleen and liver where dynamic recycling of membrane sphingolipids derived from blood cells occurs. Glycosphingolipid accumulation in engorged, often multinuclear histiocytes, generates the pathognomonic Gaucher cell [6]. With the exception of the nervous system, functional disturbances and tissue injury occur at sites where macrophages are most abundant, such as the skeleton (marrow), spleen, liver and sometimes the lung [2, 6, 8, 10]. Systemic manifestations reflect combinations of bone marrow and visceral involvement due to activation of histiocytes with local inflammatory responses; fibrosis with scarring may follow. In patients with symptomatic disease, a sustained inflammatory state is accompanied by release of cytokines and hepatosplenomegaly and in children, delayed growth and puberty [11–13]. In the absence of specific therapy, massive splenomegaly complicated by cytopenias may necessitate splenectomy. Gaucher disease may impair skeletal development with defective modelling, poor mineralization, osteolytic lesions and osteonecrosis—the latter mainly occurring at the epiphyses of long bones. Osteonecrosis may be explained by compromised blood flow in the growth plate microvasculature (Fig. 1 and Additional file 2: Fig. S1). It may be driven by glucosylceramide-related activation of macrophage-inducible C-type lectin (Mincle) with effects on osteocytes [14, 15].

The neurological complications are also not fully understood but unlike the pathology in macrophage-rich tissues, mainly reflect impaired lysosomal recycling of *endogenous* glucosylceramide derived from the membrane turnover in long-lived neural cells although perivascular accumulation of pathological storage macrophages in cerebral and cerebellar sub-cortical white matter occurs in chronic neuronopathic disease (as in Norrbottnian patients). However, neuronophagia with microglial satellitosis in the cerebral cortex and dentate nucleus occurs with biochemical and ultrastructural evidence of glucosylceramide and psychosine excess, the latter apparently correlated with neuronal loss [16, 17]. Systematic neuropathological studies after necropsy in thirteen patients classified across all three subtypes, including those with parkinsonism and dementia (with Lewy bodies containing synuclein) in type 1, identified distribution of disease uniquely affecting cerebral cortical layers 3 and 5, hippocampal CA2-4, and layer 4b of the calcarine cortex correlated with neurological impairment [18]. Prominent neuronal loss occurred in acute neuronopathic (type 2) and chronic neuronopathic disease (type 3) with progressive myoclonic encephalopathy; astrogliosis was the only neuropathological finding in ‘non-neuronopathic’ Gaucher disease.

In resource-rich countries, patients with Gaucher disease have access to transformative molecular therapies which address its systemic and skeletal manifestations. However, in many countries availability of these advanced medications is restricted by cost and by the practical arrangements associated with high-cost recombinant proteins that must be given regularly by slow infusion. Current treatments include recombinant human glucosylceramidase modified for preferential targeting to macrophages and given intravenously, or oral therapies involving small molecules with distinct modes of action [8, 19–22]. The full licensed doses for initiating enzyme therapy (60 iu/Kg every two weeks) are not universally used because of the heterogeneity and the multi-systemic nature of Gaucher disease. This is generally accepted by regulatory authorities in the context of ‘individualized’ therapy. Once the individual patient response for all relevant clinical manifestations is established, dosages and frequency of administration may be adjusted with the goal to either maintain already reached optimal parameters for all clinical manifestations or further improve those clinical parameters which have not yet been normalised. While there is a trend to lower doses in resource-poor settings, some insurers/ payers in relatively rich countries challenge costs; where monitoring is not optimal or expertise lacking, dosing may not control disease in all compartments.

Hitherto, two systemically active oral preparations, miglustat and eliglustat, based on the principle of substrate restriction have been approved for type 1 Gaucher disease. These molecules can attenuate the biosynthesis of glucosylceramide to allow the residual lysosomal digestive function to clear the pathological excess of the sphingolipid [23–29]. Targeted enzyme therapy is available for type 1 and the systemic manifestations of type 3 Gaucher disease. In the United Kingdom (UK), decisions about treatment are determined by expert physicians guided by patient preference and local prescribing rules.

The variable course of Gaucher disease and availability of specific therapies with distinct modes of action prompted us to seek a better understanding of its response to different interventions. To quantify the effects of treatment, we explored data collected during long-term real-world delivery of care over several decades of specialist practice across the UK and built a platform based on repeated measures and in-depth clinical phenotyping. With an engaged national cohort of patients attending specialist centres, we collected retrospective and prospective data in those with a confirmed clinical, biochemical and genetic diagnosis. Funded principally by the UK Medical Research Council, the Gaucher Investigative Therapy Evaluation (GAUCHERITE), enrolled 250 patients aged 5–87 years. The cohort includes patients with all but the most acute neurological forms of the disease (type 2, OMIM 230900 and the sporadic lethal perinatal form, OMIM 608013).

Once established, the skeletal and neurological features of Gaucher disease become irreversible causes of disability: hence these domains were chosen for detailed study. Here we report the clinical and radiological characteristics of the cohort in which the known determinants of outcome are recorded: these include clinical severity scores and the onset and rate of disease progression in the context of different interventions (e.g. splenectomy or molecular therapy). With extensive imaging, exploration of potential biomarkers and longitudinally repeated in-depth determinations, we have established a data-rich analytical platform further to investigate pathogenesis. To inform optimal management, the ultimate aim of this work is to define therapeutic endotypes of Gaucher disease. Combined with extensive haematological and biochemical testing, as well as imaging and DXA studies, these activities meet the criteria for deep phenotyping [30].

## Materials and methods

### Study design

In the UK, treatment for Gaucher disease is available only at specialist centres approved by the National Health Service (NHS). All patients attending each of the eight NHS specialist centres in England (Additional file 1: Table S2) were invited to participate. Clinical, radiological and laboratory data, including case report forms and other study documents, were securely recorded in the bespoke, web-based, relational GAUCHERITE database using unique identifier numbers for each centre and the individual patients attending.

Data were collected at defined intervals and transcribed from GAUCHERITE clinical study forms that include records of structured physical examinations preceded by completion of a dedicated health questionnaire. The clinical interrogation was mainly directed to skeletal events, physical manifestations and specialised neurology; comorbidities were also captured. Separately, patients or carers completed detailed quality-of-life, fatigue, health and anxiety questionnaires; specific questions were related to growth measures, as well as voice changes or breast development, as well as the age at menarche and the menopause. All treatments were recorded with emphasis on therapies directed to bone disease as well as the dosing of, and adherence to, molecular therapies administered specifically for Gaucher disease.

As set out in Additional file 1: Table S3, plasma and serum biomarkers have been measured over intervals and the data captured—including the tissue-response analytes, chitotriosidase activity and the concentration of CC chemokine pulmonary and activation-regulated chemokine (PARC)/CCL18. From the outset, a tissue biobank was developed (curated by FMP, Gaucherite Co-Principal Investigator). Biological material was collected from 238 patients (182 with serial samples) and frozen. This resource is suitable for analysis of sphingolipid biomarkers, including unacylated metabolites of glucosylceramide; genomic DNA is also retained for future analysis in ethically approved research studies.

### Study population

Two hundred and fifty patients with a confirmed diagnosis of Gaucher disease were enrolled between May 2015 to October 2017; this pre-set target, included adults and children and represents ≈ 85% of all those patients known in the UK (< 290). The database was locked in July 2018, allowing 0.5–3 years of prospective data to be collected and retrospective data from > 2–25 years. The patients included those diagnosed with the so-called non-neuronopathic (type 1) and chronic neuronopathic (type 3) disease variants. Patients likely to die within six months, those unable to understand the consent form, or those otherwise judged unsuitable by the investigator, were not recruited. These constitute a group of more than 50 known to have died since 1990 with longitudinal data also held on the database and shortly to be reported.

Definitive diagnosis of Gaucher disease was based on enzymatic assay in leukocytes or skin fibroblasts; this was supported by molecular analysis of the *GBA1* gene, except in one adult patient with a genetically confirmed diagnosis of saposin C deficiency (later treated with eliglustat). Genotyping was carried out in regional NHS diagnostic services by targeted Sanger sequencing using *GBA1* primers (Transcript: NM_000157.3) for 11 individual exons and intron boundaries using Primer3Plus to obtain PCR amplification products from all coding sequences and those in flanking intronic regions. Conventional nomenclature was used to describe *GBA1* mutations and principally employs a long-established protein designation derived from AAC63056.1 [31]; in this manuscript, when first describing mutant *GBA1* alleles, the 2016 HGVS nomenclature [32] is also used.

### Data collection

Prospective and retrospective clinical data were entered into the GAUCHERITE electronic case report form, including, for adult patients, medical records held at former paediatric specialist centres. The median number of visits was 26.1 (28.1 ± 17.4, mean ± standard deviation, SD) and median follow-up was 17.3 years, range up to 55 years (16.5 ± 9.2, mean ± SD). One child, aged 5 years, had clinical data and investigations entered on enrolment but was not further followed up during the study period. All these data including archived medical imaging studies (Additional file 1: Table S3) were anonymised. Regular data audits were conducted to ensure quality and completeness. A detailed description of the parameters collected is set out in the Additional file 1. Saccadic eye movements were measured prospectively under separate ethical approval in a consented subset of subjects using the EyeSeeCam device with a head-mounted camera for video-oculography [33, 34].

Fragility fracture was defined according to the World Health Organization [35] definition of low-impact fractures (see Additional file 1). The term “Symptomatic Osteonecrosis”, as used here, represents an event recorded in the clinical record by the treating physician as osteonecrosis or avascular necrosis and supported by a description of characteristic symptoms; radiological confirmation was not required for this purpose.

### Statistical analysis

Unless otherwise stated, baseline parameters are described at the time of enrolment into GAUCHERITE for all patients by reporting frequencies and proportions for categorical variables and summary statistics for continuous variables.

Time-to-event outcomes and repeated events data were described using Cox cumulative hazard functions and Andersen-Gill methods, respectively. Time origin for these analyses depended on the outcome and the specific research questions. Subjects were not included if relevant dates were not recorded. For the purpose of these analyses, database lock was July 31 2018. To study the effect of age at initiation of enzyme therapy or substrate reduction therapy on time from presentation with Gaucher disease to bone manifestation, we initially examined six age categories (under 10, 10–19, 20–29, 30–39, 40–49, and 50 years or older). Hazard ratios among the two younger groups and among the four older age groups was similar. Thus, for analytical purposes, age at initiation of specific treatment was stratified into two categories: younger than 20 years, and greater or equal to 20 years. Statistical significance was assessed at the 5% significance level after accounting for the possibility of model misspecification using robust standard errors; where used, 95% confidence limits are shown in parentheses. The STATA SE v14.2 and R software programme was used for all analyses.

## Results

### Population demographics and disease characteristics

Table 1 shows the baseline characteristics of the cohort: the population is stratified by age (children, up to the age of 18 years; adults, 18 years or older) and the initially assigned subtype of Gaucher disease (type 1 and 3). Of the 250 patients (128 male and 122 female, median age 46 years, range: 5–87), 223 had a clinical diagnosis of non-neuronopathic type 1 Gaucher disease (120 males and 103 females; median age 48 years, range: 5–87) and 27 of neuronopathic type 3 Gaucher disease (8 male and 19 female; median age 23 years, range 5–60), as determined by the participating physician. Of note, 26 patients (13 male and 13 female; 17 type 1 and 9 type 3) were under 18 years of age when enrolled. Of the patients with type 1 Gaucher disease, 7 had Parkinson disease (median age of this diagnosis 52 years, range: 35–70). Eighty-five patients had a family history of Parkinson disease and/or dementia.

**Table 1:** Clinical characteristics of the GAUCHERITE cohort and subgroups at recruitment

Variables	Categories*	Entire cohort (N = 250) [%]	Children (N = 26) [%]	Adults (N = 224) [%]	Gaucher Type 1 (N = 223) [%]	Gaucher Type 3 (N = 27) [%]
Gender	Male	128 [51]	13 [50]	115 [51]	120 [54]	8 [30]
	Female	122 [49]	13 [50]	109 [49]	103 [46]	19 [70]
Recruitment age (y)	Mean age ± SD	44 ± 19	11 ± 4	48 ± 16	46 ± 18	26 ± 16
	Median (range)	46 (5–87)	11 (5–17.8)	49 (18.7–87)	48 (5–87)	23 (5–60)
Jewish descent	Yes	43 [17]	1 [4]	42 [19]	43 [19]	–
	No	207 [83]	25 [96]	182 [81]	180 [81]	27 [100]
Consanguinity	Yes	18 [7]	2 [8]	16 [7]	9 [4]	9 [33]
	No	232 [93]	24 [92]	208 [93]	214 [96]	18 [67]
Age at symptom onset (y)	Mean age ± SD	16 ± 15	3 ± 2	18 ± 15	18 ± 15	3 ± 4
	Median (range)	11 (0–72)	2 (0–8)	13 (0–72)	13 (0–72)	1 (0–16)
	No symptoms	13	–	13	12	1
	Unknown	5	–	5	4	1
First symptoms	Enlarged abdomen	37 [15]	6 [23]	31 [14]	28 [12.5]	9 [33]
	Enlarged abdomen plus other symptoms	67 [27]	8 [31]	59 [26]	56 [25]	11 [40]
	Easy bleeding/bruising	34 [14]	1[4]	33 [15]	34 [15]	–
	Easy bleeding/bruising plus other symptoms	36 [14]	4 [15]	32 [14]	35 [16]	1 [4]
	Fatigue	9 [4]	–	9 [4]	9 [4]	–
	Fatigue plus other symptoms	6 [2]	–	6 [3]	6 [3]	–
	Bone disease	15 [6]	–	15 [7]	15 [7]	–
	Other non-classical GD symptoms	32 [13]	7 [27]	25 [11]	28 [12.5]	4 [15]
	No symptoms	13 [5]	–	13 [6]	12 [5]	1 [4]
	Unknown	1 [< 1]	–	1 [< 1]	–	1 [4]
Presentation age (y)	Mean age ± SD	18 ± 16	3 ± 2	20 ± 16	20 ± 16	3 ± 4
	Median (range)	13 (0–72)	2 (0–8)	16 (0–72)	16 (0–72)	2 (0–16)
	Missing age	2	–	2	1	1
Age at diagnosis (y)	Mean age ± SD	21 ± 17	4 ± 3	23 ± 17	23 ± 17	3 ± 4
	Median (range)	16 (0–73)	3 (1–10)	20 (0–73)	20 (0–73)	2 (0–16)
Diagnostic pathway	Enzyme assay	21 [8]	7 [27]	14 [6]	17 [8]	4 [15]
	GBA-gene sequencing	23 [9]	1 [4]	22 [10]	23 [10]	–
	Bone marrow histology	71 [29]	3 [11]	68 [30]	68 [30]	3 [11]
	Liver histology	12 [5]	2 [8]	10 [5]	11 [5]	1 [4]
	Spleen histology	11 [4]	–	11 [5]	9 [4]	2 [7]
	Other histology	1 [< 1]	–	1 [< 1]	1 [< 1]	–
	Combination of these methods	105 [42]	13 [50]	92 [41]	89 [40]	16 [59]
	Clinical history	5 [2]	–	5 [2]	5 [2]	–
	Unknown	1 [< 1]	–	1 [< 1]	–	1 [4]
Parkinson disease	Yes	7 [3]	–	7 [3]	7 [3]	–
	Age at diagnosis, years, mean ± SD	54 ± 12	–	54 ± 12	54 ± 12	–
	Median (range)	52 (35–70)	–	52 (35–70)	52 (35–70)	–
	No	243 [97]	26 [100]	217 [97]	216 [97]	27 [100]
Family history of Parkinson/dementia	Yes	85 [34]	4 [15]	81 [36]	76 [34]	9 [33]
	No	165 [66]	22 [85]	143 [64]	147 [66]	18 [67]
*GBA1* Genotypes	N370S/N370S	33	–	33	33	–
	L444P/L444P	20	6	14	–	20
	R463C/R463C	2	–	2	2	–
	W184R/W184R	1	–	1	1	–
	N370S/L444P	38	–	38	38	–
	N370S/other	106	11	95	106	–
	L444P/R463C	12	2	10	11	1
	L444P/other	7	2	5	4	3
	R463C/other	15	2	13	13	2
	Other /other	14	2	12	13	1
	Unknown/NA* * heterozygous *PSAP* mutations	2	1	1	2	–
Failure to thrive in childhood	Yes	65 [26]	–	65 [29]	56 [25]	9 [33]
	No	141 [56]	–	141 [63]	134 [60]	7 [26]
	Unknown/NA	44 [18]	26 [100]	18 [8]	33 [15]	11 [41]
Considered shorter than school peers	Yes	86 [35]	–	86 [38]	77 [34.5]	9 [33]
	No	123 [49]	–	123 [55]	116 [52]	7 [26]
	Unknown/NA	41 [16]	26 [100]	15 [7]	30 [13.5]	11 [41]
Considered themselves underweight	Yes	71 [29]	–	71 [32]	64 [29]	7 [26]
	No	138 [55]	–	138 [61]	129 [58]	9 [33]
	Unknown/NA	41 [16]	26 [100]	15 [7]	30 [13]	11 [41]
Delayed puberty	Yes	54 [22]	–	54 [24]	49 [22]	5 [18]
	No	148 [59]	–	148 [66]	137 [61]	11 [41]
	Unknown/NA	48 19]	26 [100]	22 10]	37 [17]	11 [41]
Onset of regular shaving/menses, N	Male	101 [79]	–	101 [88]	96 [80]	5 [63]
	Years, mean age ± SD	17 ± 2	–	17 ± 2	17 ± 2	20 ± 4
	Median (range)	16 (9–26)	–	16 (9–26)	16 (9–23)	18 (16–26)
	Missing/NA	27	13	14	24	3
	Female	100 [82]	–	100 [92]	89 [86]	11 [58]
	Years, mean age ± SD	14 ± 2	–	14 ± 2	14 ± 2	13 ± 2
	Median (range)	14 (6–19)	–	14 (6–19)	14 (6–19)	13 (11–16)
	Missing/NA	22	13	9	14	8
Menopause, N	Yes	51 [42]	–	51 [47]	48 [47]	3 [16]
	Age occurred years mean ± SD	46 ± 7	–	46 ± 7	46 ± 7	39 ± 10
	Median age (range)	47 (26–58)	–	47 (26–58)	47 (26–58)	36 (31–51)
HRT status	Yes	20 [39]	–	20 [39]	18 [38]	2 [67]
	No	29 57]	–	29 [57]	28 [58]	1 [33]
	Missing	2	–	2	2	–

*HRT* hormone replacement therapy, *NA* not applicable, *SD* standard deviation

*Categorical variables are expressed as frequency [percent]. Continuous variables are expressed as mean ± SD and median (range)

Forty-three patients (17%) stated that they were of Jewish descent; no patient of Jewish ancestry had neuronopathic (type 3) Gaucher disease. No other ethnic information was formalized for classification. Despite the rarity of the condition, overt consanguinity was identified in only 18 (7%) of the parents. Onset of symptoms or signs attributed to Gaucher disease had occurred earlier in patients with type 3 disease (median at 1 year, range: < 1–16) compared with those assigned to the type 1 category (median age 13 years, range: < 1–72). The most frequent presenting manifestation was an enlarged abdomen (15%), often with a bleeding tendency (17%). Other manifestations are set out in Table 1.

Most patients were diagnosed in childhood: the median age was 16 (range: < 1–73) years. Those with type 3 disease were much younger at diagnosis (median age 2 years, range: < 1–16) than type 1 (median age 20 years, range: < 1–73). The ratio of females to males in this group is 2.4 but does not differ significantly from a random sample (χ^2^ 1.53 with Yates correction, p = 0.216). Of note, 13 (5%) patients did not give a history of Gaucher-related symptoms: 8 were diagnosed as a result of family screening after an index case was identified; 4 were identified incidentally (test performed for a reason unrelated directly to health) and in one patient in response to other manifestations not considered to be Gaucher-related. None was identified by prenatal diagnosis or newborn screening. Although the clinical diagnosis in all cases was suspected by a combination of features and acid β-glucosidase assay is now more often requested, historically the most common primary diagnostic indicator was bone marrow biopsy (29%). Confirmation of the suspected diagnosis in all cases required either definitive measurement of enzyme activity (typically in peripheral blood leukocytes) or molecular analysis of *GBA1* in genomic DNA*.* To detect recombinant alleles and those harbouring deletions such as the del55 bp, as well as novel *GBA1* variants, sequencing of the *GBA1* locus in genomic DNA was undertaken in the entire cohort (see methods and below). The procedure followed standard clinical sequencing practice to ensure that the functional gene and not the vicinal *GBA* pseudogene was amplified for characterization in isolation.

Complete genomic sequencing identified causal mutations of the *GBA1* gene in 248 patients (Additional file 1: Table S7); one patient harboured two previously unknown mutant alleles and in one sibling of a known patient, no DNA was available for analysis; two mutant *PSAP* alleles in *trans* had been determined in one adult. The most common *GBA1* variant was p.Asn409Ser (N370S) followed by p.Leu483Pro (L444P) and p.Arg502Cys (R463C), accounting for 42.2, 19.5 and 3.8 percent of the 498 mutant *GBA1* alleles, respectively. Genotype frequencies reflected this distribution: 33 patients had a homozygous N370S genotype of whom a majority but not all stated that they were Jewish (all had non-neuronopathic type 1 disease); 20 were L444P homozygotes (all with neuronopathic disease); two patients were homozygous for R463C. Of the most frequent compound heterozygotes, 38 were N370S/L444P and 12 were L444P/R463C. Three of the seven patients with Parkinson disease were homozygous for N370S; of the other four, one was homozygous for R463C, two were N370S/L444P and one had the L444P/R463C compound *GBA1* genotype. In patients originally classified as having type 1 disease, four fifths harboured at least one copy of N370S; in those classified as type 3 disease, nearly 90% had at least one copy of the recurrent L444P allele. Both alleles were present among the 40 patients who were neurologically re-evaluated; apart from those related to Parkinson disease, no patient habouring the N370S allele was found to have neurological signs. In the cohort as a whole, recombinant, splice-site, the RecΔ5 (del55bp) and novel mutant alleles, as well as previously described double missense mutations in *cis* (e.g. D409H + H255Q) were each identified in several patients (Additional file 1: Table S7).

### Skeletal manifestations

The skeletal manifestations of Gaucher disease in this study cohort were clinically and radiologically diverse (Fig. 1, Table 2 and Additional file 2: Fig. S1). Quantification of the periodicity and presence or absence of bone pain entered into the Zimran Severity Score Index [36] was carried out at enrolment in 201 patients. Of these, 131 suffered pain (chronic in 31 patients, frequent in 34, occasional in 41, periodicity not stated in 25); pain occurred at all ages and only 70 were pain-free. The severity of skeletal pain, assessed by using the Gaucher Disease Type 1 Severity Scoring System, GD-DS3 [37], was recorded in a subset of patients (n = 129), and was extreme in two, severe in 14, moderate in 47, mild in 41, and was not scored in 25 patients.

**Table 2:** Bone manifestations and radiological characteristics of the GAUCHERITE cohort and subgroups at recruitment

Variables	Categories*	Entire cohort (N = 250) [%]	Children (N = 26) [%]	Adults (N = 224) [%]	Gaucher Type 1 (N = 223) [%]	Gaucher Type 3 (N = 27) [%]
History of Fragility fracture	Yes	37 [15]	–	37 [17]	29 [13]	8 [30]
	1 fracture	22	–	22	17	5
	> 1 fracture	15	–	15	12	3
	Age at 1st event, years, mean ± SD	38 ± 19	–	38 ± 19	42 ± 18	23 ± 16
	Median (range)	36 (4–78)	–	36 (4–78)	44 (13–78)	20 (4–50)
	No	213 [85]	26 [100]	187 [83]	194 [87]	19 [70]
Fracture site	Spine	24	–	24	20	4
	Hip/Femur	23	–	23	15	8
	Ribs	12	–	12	10	2
	Wrist	5	–	5	4	1
	Other bones	15	–	15	14	1
History of Symptomatic Osteonecrosis	Yes	76 [30]	–	76 [34]	70 [31]	6 [22]
	1 event	43	–	43	39	4
	≥ 2 events	33	–	33	31	2
	Mean age ± SD at 1st event, years	26 ± 16	–	26 ± 16	26 ± 16	14 ± 2
	Median age (range)	22 (4–64)	–	22 (4–64)	22 (4–64)	14 (12–16)
	Age unknown	4	–	4	2	1
	No	174 [70]	26 [100]	148 [66]	153 [69]	21 [78]
Symptoms of Osteonecrosis event	Yes	76 [30]	–	76 [34]	70 [31]	6 [22]
	Classical acute bone crisis	30	–	30	27	3
	Pain	45	–	45	42	3
	Symptomatic, other than pain	1	–	1	1	–
Presence of bone/joint pain	Yes	131 52]	4 [15]	127 [57]	118 [53]	13 [48]
	Continuous/chronic	31	–	31	27	4
	Frequently	34	1	33	31	3
	Occasional	41	3	38	37	4
	Pain periodicity not recorded	25	–	25	23	2
	No	70 [28]	13 [50]	57 [25]	61 [27]	9 [33]
	Unknown	49 [20]	9 [35]	40 [18]	44 [20]	5 [19]
Severity of bone/joint pain (GD1–DS3 score)	Yes	129 [52]	4 [15]	125 [56]	116 [52]	13 [48]
	Extreme	2	–	2	2	–
	Severe	14	–	14	13	1
	Moderate	47	1	46	41	6
	Mild	41	1	40	39	2
	Pain severity not recorded	25	2	23	21	4
	Unknown	121 [48]	22 [85]	99 [44]	107 [48]	14 [52]
Lytic lesions	Yes	13 [5]	–	13 [6]	11 [5]	2 [7]
	No	237 [95]	26 [100]	211 [94]	212 [95]	25 [93]
Osteoarthritis	Yes	105 [42]	–	105 47]	100 [45]	5 [19]
	No	145 [58]	26 [100]	119 [53]	123 55]	22 [81]
Orthopaedic procedure	Yes	62 [25]	–	62 [28]	55 [25]	7 [26]
	No	188[75]	26 [100]	162 [72]	168 [75]	20 [74]
*DXA*						
Spine	Number available	189	15	174	171	18
	BMD, g/cm^2^, mean ± SD	0.984 ± 0.150	0.836 ± 0.189	0.997 ± 1.139	0.989 ± 0.144	0.937 ± 0.196
	Median (range)	0.982 (0.547–1.446)	0.759 (0.547–1.151)	0.986 (0.695–1.446)	0.982 (0.603–1.446)	0.989 (0.547–1.248)
Total Hip	Number	165	3	162	152	13
	BMD, g/cm^2^, mean ± SD	0.930 ± 0.153	0.716 ± 0.133	0.935 ± 0.151	0.934 ± 0.153	0.892 ± 0.159
	Median (range)	0.924 (0.600–1.276)	0.687 (0.600–0.861)	0.926 (0.607–1.276)	0.923 (0.607–1.276)	0.874 (0.600–1.253)
Femoral Neck	Number	165	3	162	152	13
	BMD, g/cm^2^, mean ± SD	0.813 ± 0.152	0.615 ± 0.120	0.816 ± 0.151	0.814 ± 0.153	0.793 ± 0.152
	Median (range)	0.801 (0.464–1.360)	0.640 (0.485–0.719)	0.804 (0.464–1.360)	0.804 (0.464–1.360)	0.789 (0.485–1.061)
Forearm	Number	77	–	77	70	7
	BMD, g/cm^2^, mean ± SD	0.658 ± 0.103	–	0.658 ± 0.103	0.660 ± 0.104	0.644 ± 0.090
	Median (range)	0.653 (0.458–0.871)	–	0.653 (0.458–0.871)	0.654 (0.458–0.871)	0.632 (0.513–0.778)

*BMD* bone mineral density, *DXA* Dual-energy X-ray absorptiometry, *GD1-DS3* Gaucher disease type 1 severity scoring system, *SD* standard deviation

*Categorical variables are expressed as frequency [percent]. Continuous variables are expressed as mean ± SD and median (range)

Symptomatic osteonecrosis, the most clinically significant and disabling skeletal manifestation of Gaucher disease, occurred in seventy-six patients. Among these, 43 had one episode and 33 had two or more episodes. Osteonecrosis caused bone pain in 45 or classical acute bone crisis in 30 cases, while only one patient had skeletal symptoms that were not painful. The median age at first episode of osteonecrosis in these 76 patients was 22 (range 4–64) years: of these, nearly 90% occurred in the femur (distal or proximal), 4% occurred in the humeral head, 3% in the tibia and 4% at other sites.

Skeletal involvement in Gaucher disease includes reduced bone mineral density (BMD) which can be detected using dual-energy X-ray absorptiometry (DXA) and predisposes to fracture. Although most patients had had multiple DXA measurements between presentation and recruitment to the study, DXA determinations ± 24 months from enrolment were available in a majority (n = 189 spine; n = 165 hip; n = 77 forearm; Table 2). Overall median vertebral bone mineral density of L1–L4 was 0.982 (range 0.547–1.446) g/cm^2^. The median total hip and femoral neck BMD were 0.924 (0.600–1.276) and 0.801 (0.464–1.360) g/cm^2^, respectively. Median forearm density was 0.653 (0.458–0.871) g/cm^2^. Among postmenopausal women and men aged 50 years and older, 24 had DXA T-scores within the healthy reference range, while 34 had T-scores between − 1 and − 2.5 that are considered osteopaenic and 23 had a T-score of -2.5 or lower—taken to indicate osteoporosis. Among premenopausal women and men younger than 50 years, 94 had DXA Z-scores within the expected range for age, while 17 had a Z-score of -2.0 or lower and thus below the expected range for age.

Sixteen males and 21 females had a history of one or more fragility fractures at any site; the median age at first fragility fracture was 36 (range 4–78) years. The spleen had been removed in twenty-three of the 37. Of the total of 79 fragility fractures at recruitment, 30% had occurred in the spine; 29% in the hip/femur; 16% were in the ribs; 6% in the wrist and 19% occurred elsewhere. As detailed in Table 1, patients with type 3 disease generally had lower bone density at a younger age: those who suffered at least one fragility fracture, had their first episode earlier (median age 20 years, range: 4–50) than those with type 1 disease with one or more fragility fractures (median age 44 years, range: 13–78 years).

Radiological signs of osteoarthritis and frank osteolytic lesions were reported in 105 and 13 patients, respectively. Erlenmeyer flask deformity based on individual radiographic assessment was present in 80 out of the 125 adult patients for whom radiographs of the knee and distal femur were available for re-analysis. A defining ratio between the diameter of the femoral shaft 4 cm from the physis to the diameter of the physeal baseline of greater than 0.57 was used [38].

Orthopaedic surgery had been undertaken in sixty-two patients: procedures were mainly carried out for joint destruction related to osteonecrosis but surgery was also required for fractures.

### Neuronopathic disease

Of the 27 patients with a diagnosis of chronic neuronopathic disease (type 3) at enrolment, the median age was 23 years (range: 5–60); four were older than 40 years. Neuronopathic features had often been detected after Gaucher disease was diagnosed: the most common presenting feature was a horizontal saccadic eye movement defect, typical of the disorder. After Gaucher disease had been diagnosed, formal recognition of the neuronopathic manifestations occurred within one year in 17 of the 27 patients, but in the remaining 10, these manifestations remained undocumented for between 2 and 48 years. Hepatosplenomegaly was the most frequent presenting manifestation, often in association with neurological signs, or as an incidental finding during assessment for an unrelated childhood illness, typically a respiratory complaint. In four patients, splenomegaly without clinical enlargement of the liver led directly to the diagnosis. One patient with neuronopathic Gaucher disease was identified after the diagnosis had been made in a sibling. In all four patients for whom longitudinal growth data were available, height velocity improved after enzyme infusions started; catch-up growth was accompanied by salutary changes in peripheral blood counts, hepatosplenomegaly and plasma chitotriosidase activity.

Two patients with neuronopathic Gaucher disease had received a bone marrow transplant as a primary intervention at the ages of 18 months and 11 years; the other patients are treated by enzyme therapy. Enzyme therapy was started at various times – before the age of 1 year to 41 years of age; median 3 years.

Impaired horizontal saccadic eye movement was present in all patients entering the study with neuronopathic Gaucher disease; more than 70% also had defective vertical saccadic movements. Strabismus was present in 22 (> 80%) of the patients (reflecting abducens nerve palsy); increased muscle tone and tendon reflexes were noted in 18 and intention tremor in 14 patients. Myoclonus and epilepsy, including myoclonic seizures, occurred in five patients. Disease severity, as determined by the modified Severity Scoring Tool (mSST) [39, 40], ranged from 0.5 to 19.5 at recruitment (higher scores indicating more severe disease; maximum 36); the mean score was 6.3 ± 5.4.

Patients with neuronopathic Gaucher disease at recruitment had non-neurological comorbidities. The most frequent were kyphosis (and/or scoliosis) and infiltrative lung disease—some patients had recurrent respiratory symptoms in the absence of a formal diagnosis of pulmonary infiltration. Of note, no patients were homozygous for the p.Asp448His (D409H) mutation in *GBA1* and, as expected, none had the associated calcific cardiac valve disease nor aorto-coronary involvement.

### Other neurological manifestations

**Fig. 2:**
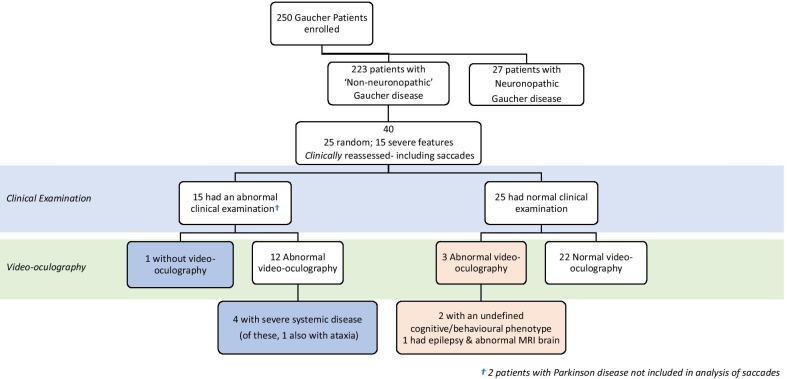
Neurological signs in Gaucher disease type 1. At enrolment, 223 of 250 patients had been classified as non-neuronopathic type 1 Gaucher disease and 27 with neuronopathic type 3 Gaucher disease. During the prospective study period, forty patients originally assigned to the type 1 disease category were subject to re-examination by a neurologist who found central nervous system signs in 15, of whom 13 had type 3 disease with clinically evident saccadic abnormalities. Of the 25 patients without clinical neurological signs, based on oculography and other clinical features, only 3 meet the criteria for reclassification as type 3 disease (X^2^ test with Yates correction, p-value < 0.00001)

Beyond the 27 patients assigned to type 3 (neuronopathic) disease at baseline as above, during the prospective study period, additional patients with neurological signs were found (see Fig. 2). Forty patients previously classified as type 1, non-neuronopathic Gaucher disease at enrolment, were examined opportunistically by a neurologist as a convenience sample. This supplementary examination included the assessment of saccadic eye movements as part of a parallel study and in all but one, a video-oculographic assessment was undertaken using EyeSeeCam [41]. Twenty-five patients were selected at random and 15 were examined because of clinical features of severe systemic disease and/or with *GBA1* genotypes recognised to be adverse: L444P, R463C, W184R in homozygous or heterozygous form as well as rare alleles (including the RecNciI complex variant). Of note, several of these potentially adverse genotypes are also present in the remaining Gaucherite cohort not yet subject to independent re-examination by a neurologist.

As depicted in Fig. 2, of the forty patients in all, 25 had a *normal* clinical examination on review and of these, three had abnormal saccades detected by oculography and on careful scrutiny, other neurological features (two had an undefined cognitive and behavioural phenotype; the remaining patient suffered seizures with abnormal magnetic resonance brain imaging). Also as shown in the figure, fifteen of the forty re-examined patients had *abnormal* neurological signs—two had Parkinson disease. Development of Parkinson disease, with or without Lewy body dementia, is an uncommon but now recognised feature of type 1 Gaucher disease; abnormal oculography occurs in Parkinson disease [42]. The thirteen (non-Parkinsonian) patients with neurological signs, including abnormal ocular signs, may be eligible for reassignment to Gaucher disease type 3, since the presence of supranuclear ophthalmoplegia with abnormal saccades is claimed to be essential for the diagnosis of chronic neuronopathic Gaucher disease [43]. Four of these patients, were found to have florid manifestations of type 3 disease with additional severe systemic features that included kyphosis.

In summary, of the group of forty patients formerly classed as having type 1 disease who were later reviewed by a neurologist in this study, sixteen were found to have neurological signs associated with neuronopathic Gaucher disease and two had Parkinson disease. Individual *GBA*1 genotypes in these patients, with totals in parenthesis, were: L444P/R463C (4); R463C/RecNci1 (4); L444P/P266R (1); L444P/P266A  (1); R463C/IVS2 + 1G (1); R463C/G377R (1); R463C/R257Q (1); R463C/R496C (1); RecNci1/ R262G (1); H311R/R359Q (1) (Additional file 1: Table S7).       

### Other manifestations

#### Haematological findings

Within 30 days of enrolment, the mean haemoglobin was 139 g/L (range 101–178) and the platelet count was 189 × 10^9^/L (range: 39–489). The effect of splenectomy on the platelet count was evident in the splenectomised subgroup compared with non-splenectomised patients; there was no material difference in haemoglobin concentration (Table 3).

**Table 3:** Laboratory characteristics of the GAUCHERITE cohort and subgroups at recruitment

Variables	Categories*	Entire cohort (N = 250)	Children (N = 26)	Adults (N = 224)	Gaucher Type 1 (N = 223)	Gaucher Type 3 (N = 27)
Haemoglobin	Number	211	11	200	193	18
	g/L, mean ± SD	139.5 ± 13.9	133.7 ± 11.6	139.9 ± 14.0	140.3 ± 13.6	131.3 ± 15.2
	Median (range)	139 (101–178)	131 (118–151)	139 (101–178)	139 (101–178)	131 (103–160)
With spleen	Number	157	11	146	146	11
	g/L, mean ± SD	140 ± 15	134 ± 12	141 ± 15	141 ± 14	129 ± 17
	Median (range)	139 (101–178)	131 (118–151)	140 (101–178)	140 (101–178)	131 (103–160)
Without spleen	Number	54	–	54	47	7
	g/L, mean ± SD	137 ± 11	–	137 ± 11	138 ± 11	135 ± 14
	Median (range)	138 (116–163)	–	138 (116–163)	138 (116–163)	131 (120–153)
Platelet count	Number	208	11	197	190	18
	10^9^/L, mean ± SD	200 ± 74	229 ± 62	198 ± 74	196 ± 74	244 ± 52
	Median (range)	189 (39–489)	213 (151–337)	187 (39–489)	184 (39–489)	234 (147–337)
With spleen	Number	155	11	144	144	11
	10^9^/L, mean ± SD	178 ± 56	229 ± 62	174 ± 54	173 ± 53	242 ± 58
	Median (range)	175 (39–337)	213 (151–337)	175 (39–321)	175 (39–321)	230 (147–337)
Without spleen	Number	53	–	53	46	7
	10^9^/L, mean ± SD	263 ± 82	–	263 ± 82	265 ± 87	248 ± 46
	Median (range)	253 (87–489)	–	253 (87–489)	253.5 (87–489)	236 (197–318)

*SD* standard deviation

*Continuous variables are expressed as mean ± SD and median (range).

#### Pulmonary disease

Pulmonary manifestations of Gaucher disease have been described but there is a lack of consensus about the nature of the pathological process and its classification. Eight patients with type 1 disease had pulmonary disease for which no other cause could be identified: thoracic imaging in this group, revealed bronchiectasis, fibrosis, interstitial disease (with nodules and interlobular septal thickening) and signs of pulmonary hypertension. Twenty-two patients with type 3 disease had intermittent respiratory symptoms: eight had recurrent respiratory infections or symptoms suggesting asthma; they had not undergone more detailed radiological investigation (e.g. computerized X-ray tomography). One patient had isolated pulmonary hypertension of unknown cause and the 13 remaining had bronchial and interlobular septal thickening, reticular-nodular shadowing with a ground-glass appearance on tomographic scans; cystic changes also occurred. One patient had recurrent pneumothoraces: histology of pulmonary tissue and bronchoalveolar lavage from this patient showed Gaucher cell infiltration. A further patient suffered pulmonary haemorrhages that required therapeutic embolisation. See Table 4.

**Table 4:** Other manifestations of the GAUCHERITE cohort and subgroups at recruitment

Variables	Categories*	Entire cohort (N = 250) [%]	Children (N = 26) [%]	Adults (N = 224) [%]	Gaucher Type 1 (N = 223) [%]	Gaucher Type 3 (N = 27) [%]
Gallstones	Yes	79 [32]	1 [4]	78 [35]	75 34]	4 [15]
	Age at diagnosis, years, mean ± SD	41 ± 13	16 ± 0	42 ± 12	42 ± 12	35 ± 19
	Median (range)	40 (11–66)	16 (16)	40 (11–66)	40 (11–66)	35 (16–55)
	Age unknown	5	–	5	5	–
	No	171 [68]	25 [96]	146 [65]	148 [66]	23 [85]
Path to diagnosis of gallstones	Incidental	43 [54]	1 [100]	42 [54]	40 [53]	3 75]
	Symptoms prompted investigations	32 [41]	–	32 [41]	31 [41.5]	1 [25]
	Unknown	4 [5]	–	4 [5]	4 [5.5]	–
Cholecystectomy	Yes	37 [15]	1 4]	36 [16]	35 [16]	2 [7]
	Age at cholecystectomy, years,	39 ± 13	16 ± 0	39 ± 12	39 ± 12	35 ± 28
	mean ± SD					
	Median (range)	37 (16–69)	16	37 (18–69)	37 (18–69)	35 (16–55)
	Missing age	1	–	1	1	–
	No	213 [85]	25 [96]	188 [84]	188 [84]	25 [93]
Cancer	Yes	23 [9]	–	23 [10]	23 [10]	–
	Haematological	4	–	4 (17%)	4 (17%)	–
	Non-haematological	19	–	19 (83%)	19 (83%)	–
	Age at diagnosis, years, mean ± SD	59 ± 15	–	58 ± 15	59 ± 15	–
	Median (range)	62 (18–83)	–	62 (18–83)	62 (18–83)	–
	No	227 [91]	26 [100]	201 [90]	200 [90]	27 [100]
Hepatic Disease	Yes	14 [6]	–	14 [6]	9 [4]	5 [19]
	Age at diagnosis, years, mean ± SD	40 ± 17	–	40 ± 17	47 ± 12	27 ± 18
	Median (range)	41 (6–75)	–	41 (6–75)	45 (37–75)	24 (6–55)
	No	236 [94]	26 [100]	210 [94]	214 [96]	22 [81]
Pulmonary disease	Yes	29 12]	8 31]	21 [9]	7 [3]	22 [81.5]
	Age at diagnosis, years, mean ± SD	19 ± 22	5 ± 2	24 ± 24	39 ± 28	12 ± 15
	Median (range)	8 (1–72)	5 (2–9)	14 (1–72)	51 (4–72)	6 (1–57)
	No	221 [88]	18 [69]	203 [91]	216 [97]	5 [18.5]

*SD* standard deviation

*Continuous variables are expressed as mean ± SD and median (range)

#### Hepatic disease

Clinical features of liver disease with abnormal imaging with or without histopathological findings indicating fibrosis or cirrhosis not clearly attributable to fatty infiltration or alcoholic liver disease, were identified in fourteen patients (9 with type 1, and 5 with type 3 Gaucher disease; Table 4). Eleven of these had been splenectomised and in several, the liver injury had been documented before enzyme therapy was started. In these 14 patients, the median interval between diagnosis of liver disease and Gaucher-specific therapy was 5.5 years but with a wide range—treatment having started 27 years before and up to 8 years after liver disease was recognized. Liver transplantation had been carried out in two patients: one had acute hepatic decompensation and the other, hepato-pulmonary syndrome. These individuals continue to receive enzyme therapy with good effect and were alive 19 and 21 years after transplantation. A further fourteen patients had structural liver abnormalities identified on imaging but a formal diagnosis by biopsy has not been made. The abnormalities include irregularity of the hepatic capsule, focal lesions, atrophy, calcification, and hepatic infarcts. More detailed studies on the hepatic and pulmonary manifestations of patients in the GAUCHERITE cohort will be reported separately and where possible this will include a systematic re-evaluation of the neurological findings across the entire cohort.

#### Malignant disease

Twenty-three patients with type 1 disease (of whom 9 were splenectomised) had documented cancer (median age 62 years, range: 18–83). Of these, > 80% had non-haematological cancer [7 cutaneous, 4 gastro-intestinal, 2 breast, 2 nervous system (cerebral meningioma and spinal cord tumour), one each of hepatic, renal, genito-urinary tract and respiratory]. Of the four patients with haematological cancer, 3 had myeloma and one, B-cell lymphoma—Tables 4, 5 and 6.

**Table 5:** Splenectomy and non-haematological cancer

Group	Non-haematological cancer	Total size	Proportion*	P-value^†^
With spleen	10	188	0.053	0.03
Without spleen	9	62	0.145	
Total	19	250		

*Proportion = Non-haematological cancer/Total size

^†^Fisher's Exact test P-value

**Table 6:** Splenectomy and haematological cancer

Group	Haematological cancer	Total size	Proportion*	P-value^†^
With spleen	4	188	0.021	0.58
Without spleen	0	62	0	
Total	4	250		

*Proportion = Haematological cancer/Total size

^†^Fisher's Exact test P-value

### Treatment status

#### Splenectomy

Overall, one quarter of the patients had undergone splenectomy (median age of the procedure, 17 years, range: 1–58). The spleen has been removed in one third of those classified with type 3 Gaucher disease—in all instances in infancy and childhood (median age 5 years, range: 1–15). Three quarters of the splenectomies had been carried out before 1992–3, when tissue-derived enzyme therapy first became available in the European region before full regulatory approval: alglucerase was marketed from 1994 in Europe; the recombinant product, imiglucerase, was approved for marketing in 1997, followed by velaglucerase alfa in 2010 [20, 44].

It is noteworthy that of the 15 patients who underwent splenectomy after 1993, cytopenia was an unusual indication. In eight patients, the spleen had been removed for diagnostic purposes (e.g. suspected malignancy) before referral to the specialist service; in a further three, the indication post-traumatic splenic rupture. Splenectomy was carried out for other reasons in three patients: inadequate responses to enzyme therapy with concern about bleeding risk, management of splenic infarction—and for an unknown indication in one patient.

#### Haematopoietic stem-cell transplantation

Two patients (1 man aged 46 and 1 woman, aged 30 years at recruitment) in the study had undergone haematopoietic stem cell transplantation as a primary intervention for Gaucher disease. Both share the p.Leu483Pro (L444P) homozygous genotype and have a classical “Norrbottnian” type 3 phenotype [6, 8, 45]. Transplantation from HLA-matched related donors was carried out in childhood (aged 11 years and 18 months, respectively). Splenectomy was undertaken before the procedure: growth and haematological parameters responded favourably—although kyphosis and neurological signs have slowly progressed in these patients. These surviving patients are among six transplanted in UK as originally pioneered by Hobbs and colleagues [46, 47].

#### Enzyme and substrate-reduction therapies

Two hundred and forty-three patients were receiving specific treatment for Gaucher disease within 6 months of recruitment to the study: velaglucerase alfa was given to 141 patients, imiglucerase to 89, miglustat to 4 and eliglustat to 7 (Additional file 1: Table S6). At the time of writing, 65 patients are taking eliglustat and will be the subject of follow-up studies related to safety, tolerability and efficacy based on this cohort. Three patients were taking enzyme therapy combined with substrate-reduction therapy: velaglucerase alfa and miglustat in a patient with type 1 disease, and imiglucerase with eliglustat in two patients with type 3 Gaucher disease.

#### Treatment-related adverse events

Sixty-eight treatment-related adverse events (of which 36 were in patients receiving miglustat) had been reported in 43 patients. None of the reactions was life-threatening. As a result of adverse events, 25 users of miglustat had stopped this treatment. Seven patients receiving enzyme therapy stopped temporarily (1–4 months) and one permanently, switching to oral therapy (miglustat for 25 months and then eliglustat after a further 53 months). The six other patients all returned to enzyme therapy, one initially returned to alglucerase (in 1993) and two patients receiving taliglucerase alfa returned to either imiglucerase or velaglucerase. These latter are the only preparations currently approved in the UK. In the cohort, infusion reactions have been recorded in two patients: one developed migraine and having developed sensitivity to all three recombinant enzyme preparations, the other switched to oral therapy (eliglustat) after study lock.

#### Bone specific therapies

Seventy-five patients had received anti-resorptive and/or anabolic drugs before recruitment. Within 6 months of enrollment, 94 patients in all had received a medication to treat or prevent skeletal disease (i.e. supplemental calcium and/or vitamin D, anti-resorptive or anabolic drugs). Seventy-nine patients had received calcium alone or in combination with vitamin D; 14 had received bisphosphonates alone or in combination with calcium and/or vitamin D supplements and one patient had received anabolic treatment in combination with calcium and vitamin D supplements (Additional file 1: Table S6).

#### Effect of splenectomy on skeletal manifestations

**Fig. 3:**
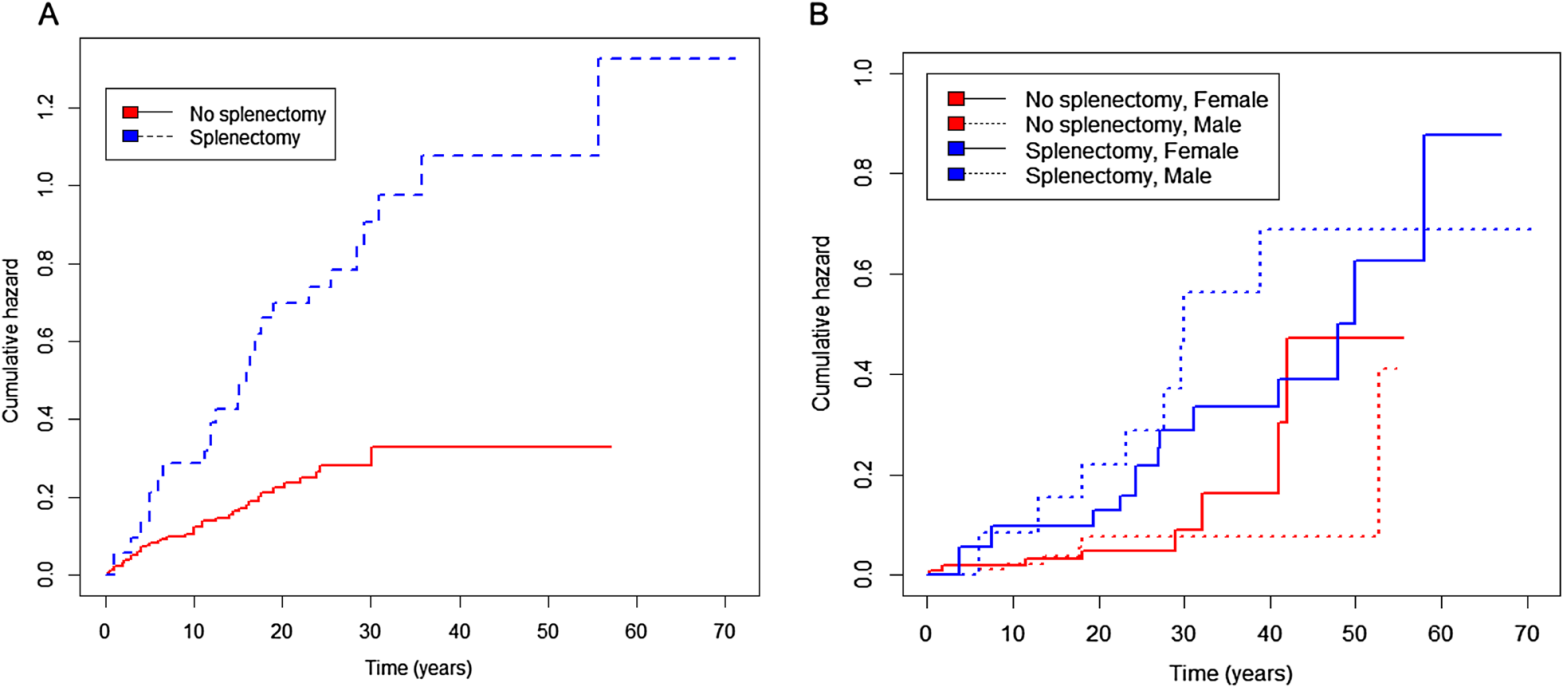
Cumulative hazard for first osteonecrosis event and fragility fracture. Controlling for gender, the hazard or risk of having a first osteonecrosis event (**A**) and first fragility fracture (**B**) after presentation of Gaucher disease was greater in patients who had splenectomy (hazard ratio of 3.32 [95% CI 1.74–5.00; p < 0.001] and 2.83 [95% CI 1.33–5.99; p = 0.01], respectively). Horizontal axis shows years after presentation with Gaucher disease

The occurrence of symptomatic osteonecrosis was strongly associated with splenectomy (Fig. 3A): controlling for gender, the hazard/intensity ratios for first and subsequent events were about 3 times greater in patients after splenectomy (p < 0.0001).

Splenectomy also influenced fracture risk (Fig. 3B). Controlling for gender, the hazard ratio for first fragility fracture in patients after undergoing splenectomy was nearly three-fold greater than those in whom the spleen was, or is intact (p = 0.01). Splenectomy was additionally associated with an approximately sixfold greater risk of repeated fragility fractures (p < 0.0001). Figure 3 shows the temporal relationship to first skeletal events and the association of splenectomy, from diagnosis onwards: the two panels compare events for osteonecrosis and fragility fracture—depicting the interaction of age and gender in fragility fractures, in which a component risk due to the menopause in women would be predicted. Risk of osteolytic lesions and the occurrence of orthopaedic procedures were about 2.7- and twofold respectively higher after splenectomy, when controlled for gender (p = 0.12 and p = 0.01, respectively).

#### Symptomatic osteonecrosis and fracture in relation to Gaucher-specific therapy

**Fig. 4:**
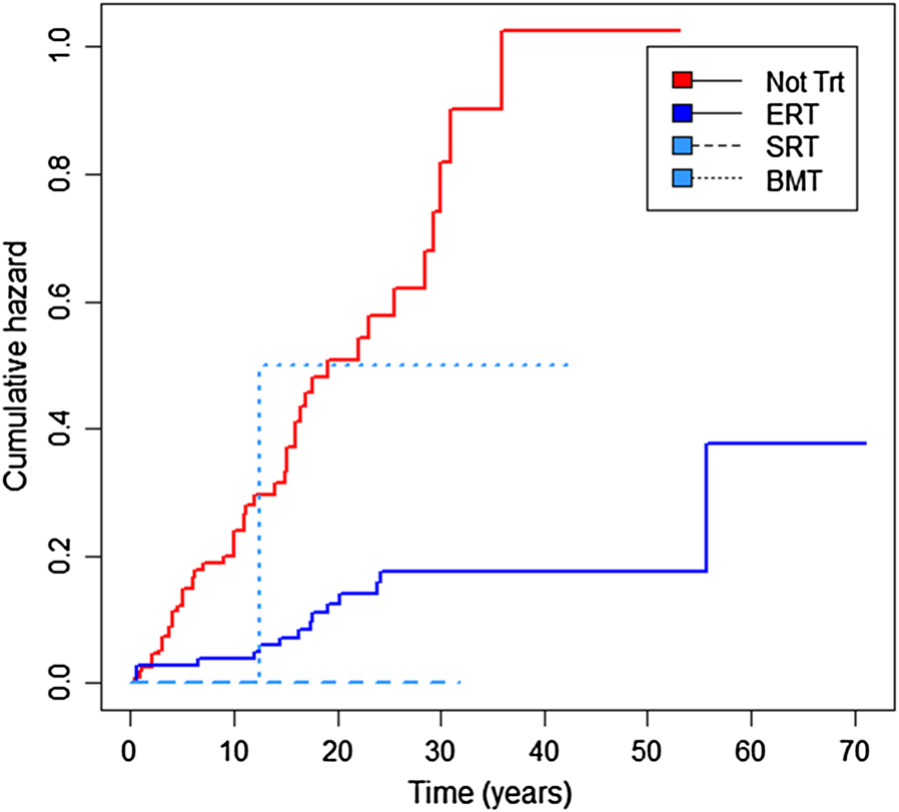
Cumulative hazard for first osteonecrosis event according to treatment status. The hazard or risk of having a first osteonecrosis event after presentation of Gaucher disease was significantly lower after starting enzyme replacement therapy (ERT) than before (hazard ratio of 0.20; 95% CI 0.11–0.38; p < 0.001). The effects of substrate reduction therapy (SRT) and bone marrow transplantation (BMT) was difficult to assess owing to the low sample size (nine and two patients received SRT or BMT as first treatment, respectively). Of the two patients with BMT, one had their first episode of osteonecrosis after the procedure. Horizontal axis shows time in years after presentation of Gaucher disease

Data from two hundred and forty-six patients were available for analysis of the relationship between the skeletal events and introduction of molecular therapies for Gaucher disease. Fifty-one first episodes of symptomatic osteonecrosis were documented in 242 patients when they were not receiving Gaucher-specific therapy (2136 person-years of follow-up), whereas 13 first events occurred in 180 patients after treatment, which was initially enzyme therapy (2493 person-years of follow-up), hazard ratio 0.2, p < 0.001. At the time of data lock, too few patients have been exposed to the other specific interventions (marrow transplantation or substrate reduction therapy) for such analysis (Fig. 4). Age at which treatment began had no detectable effect.

In an Andersen-Gill analysis of 120 repeated events, the rate of symptomatic osteonecrosis was also reduced after specific treatment was started (hazard ratio 0.45, p < 0.001) and again there was no age effect. In contrast, neither the time to first fragility fracture nor hazard of repeated fragility fractures was associated with any, or all of the combined specific treatments, nor was there an effect of age at which treatment started.

#### Skeletal complications persist in patients receiving bone-specific treatment

Males with Gaucher disease who had treatment given to improve bone density, on average had a lower fragility fracture rate (p < 0.05) and incidence of lytic lesions (p < 0.01) compared with females receiving bone modifying treatment. When followed over 15 years, the average fracture rate for males was 14.8 (95% confidence interval 6.8 to 32.6) per 100 person-years; the corresponding rate for females was 63.5 (43.5 to 92.7).

## Discussion

In the group of more than eighty inborn lysosomal disorders, Gaucher disease is unique in its susceptibility to treatments and with sustained investment, the transformative efficacy of molecular therapies sets this ultra-rare disorder alongside haemophilia and cystic fibrosis in the Pantheon of orphan diseases. Biotechnological innovation has generated a diverse selection of drugs with distinct modes of action and indications. The competitive success of these ventures and marketing exclusivity of high-cost orphan drugs, mandate the need for independent therapeutic research that includes real-world data.

As in other advanced economies, most patients with Gaucher disease in the United Kingdom have received specific treatment but in the adults reported here, access to molecular therapies was often deferred until long after diagnosis. As a consequence, irreversible injury, especially to the skeleton and larger joints, became an inevitable burden. To appraise new treatments for this heterogeneous and chronic condition, powerful systems to collate, hold and comprehensively analyse extensive long-term data are needed.

GAUCHERITE is a platform concept with a national cohort of patients that seeks (1) to determine clinical burden; (2) characterize the clinical manifestations by in-depth phenotyping and (3) ultimately optimize management by therapeutic stratification of Gaucher disease. We focused principally on the skeletal aspects but also indicate the potential importance of timely recognition of neuronopathic features, which are associated with adverse progression of the bone disease. While no effective therapy for the neurological manifestations is as yet available, molecular therapies have salutary systemic effects  which include manifestations in bone: nonetheless, persistent bone pain and disabling skeletal complications remain dominant  unmet needs in  many patients [48].

Our study is aligned to current stratification methodology [30]: use of longitudinal data of sufficient depth enables the effects of orphan drugs to be analysed even in a rare disease. Our approach has been validated, since patients were readily stratified in respect of two broad interventions: (1) splenectomy, a palliative measure to reduce the risk of bleeding and infection due to pancytopenia, and (2) introduction of molecular therapies.

In the era before specific therapies became available, splenectomy was required to ameliorate severe hypersplenism with the risk of life-threatening haemorrhage: however the procedure is strongly associated with recurrent osteonecrosis and, as shown here, also fragility fractures. Post-splenectomy fractures occur with markedly different trajectories according to gender—attributable to additional effects of osteoporosis at the menopause. The procedure signals a clinically distinct subgroup of patients with Gaucher disease with a high demand for orthopaedic services. As to cause and effect, we note that a forerunner of this study conducted in the UK population, identified a temporal relationship of osteonecrosis to the immediate years after splenectomy, indicating a causal relationship [49–54].

Mechanistic explanations for the diverse consequences of splenectomy include: (1) after the procedure, a greater burden of glycosphingolipids derived from the destruction of blood cells will fall on liver macrophages (Küpffer cells) and bone marrow. Greater encroachment by pathological macrophages will also induce greater local pro-inflammatory cytokine and chemokine release. We suggest that this will have adverse effects on the capillary network at junctional zones related to the growth plate and enhance the pathological accumulation of β-glucosylceramide a key ligand, of the mincle protein in macrophages and related osteoclasts [14, 15]. Poor perfusion will increase the risk of local ischaemia to the bone marrow; (2) splenectomy has multiple effects on the formed elements of the blood and increased viscosity and platelet aggregation index induces a thrombotic tendency [55]. Increased platelet numbers consequent upon the abrupt decrease in splenic pooling may also increase the risk of osteonecrosis; splenectomy is likely to amplify the changes in red-cell deformability due to alterations in the lipid composition of red-cell membranes [56]. Finally, increased red-cell rigidity will potentiate the effect of removing the spleen on the marrow compartment, since by analogy with sickle-cell disease, it will impair the deformability and flow properties of red cells within the microcirculation at watershed regions of oxygen and nutrient supply in long bones [57].

The greatly increased hazard of fragility fractures showed an incident pattern over time that differs between men and women: neither phenomenon has been reported. Although splenectomy in Gaucher disease is known to be associated with low bone mineral density [58], in this respect it  resembles the effect of hyposplenism in sickle-cell anaemia, in which bone mineral density is greatly reduced [59]. Here we show a strong association with fragility fractures (Table 7 and Fig. 3). Of the 62 patients lacking a spleen, 21 had sustained multiple fragility fractures and 38 had developed osteonecrosis; these data compare with 16 and 38 of 188 Gaucher patients who had intact spleens, respectively. We argue that the link with the three-fold increased hazard of fragility fractures identified here is clinically important. Since completion of our analysis, investigation of an international registry of type 1 Gaucher patients has recently reported a multivariate analysis of fractures in three age cohorts among patients with known splenectomy status and at least one skeletal assessment on treatment (3216 of 6422 patients) [60]. Of note, a greater proportion of those splenectomized before enzyme treatment (with alglucerase or imiglucerase), had fractures (56% greater risk of fracture post-splenectomy). The authors state that for patients aged 50 years or older when the therapy was first given, the analysis may have been affected by the exclusion of those who had suffered fractures during the long interval between the date of splenectomy and starting enzyme. Nonetheless in their  report, the fracture risk attributed to the procedure was found only statistically significant (p = 0.039) in adults aged between 18 and 50 years at the time enzyme treatment started.

**Table 7:** Impact of splenectomy on skeletal manifestations since presentation of Gaucher disease

		Hazard ratio	P-value	[95% CI]	
1st fragility fracture	Splenectomy	2.83	0.01	1.33	5.99
N = 247	Gender effect	1.04	0.91	0.52	2.07
Multiple fragility fractures*	Splenectomy	5.78	<0.0001	2.32	14.42
N = 247	Gender effect	0.59	0.17	0.28	1.24
1st osteonecrosis	Splenectomy	2.95	<0.0001	1.74	5.00
N = 246	Gender	0.80	0.37	0.48	1.31
Osteonecrosis events*	Splenectomy	3.32	<0.0001	1.98	5.56
N = 246	Gender effect	0.95	0.83	0.59	1.53
Presence of Erlenmeyer flask deformity	Splenectomy	0.98	0.95	0.59	1.64
N = 248	Gender effect	0.80	0.34	0.50	1.27
Presence of lytic lesions	Splenectomy	2.70	0.12	0.76	9.52
N = 248	Gender effect	0.26	0.08	0.06	1.16
Orthopaedic surgery	Splenectomy	1.99	0.01	1.15	3.44
N = 244	Gender effect	0.84	0.52	0.50	1.41

Origin: Gaucher disease presentation

*Event rate was estimated using Andersen-Gill method.

The cause is not obvious and may either be a direct or indirect consequence of the procedure. We propose that rapidly progressive inflammatory disease with indications for early splenectomy—especially in young patients not promptly diagnosed and thus not optimally treated—may be the explanation. Florid Gaucher disease activity in childhood is a chronic inflammatory state that stunts growth and delays puberty—not only is modelling impaired with manifest osseous disease as above, but the achievable peak mass of bone is also decreased.

Splenectomy has potential consequences for the skeleton beyond osteonecrosis, risk of fracture and, possibly a trend towards more frequent osteolytic lesions. Since the risk of requiring orthopaedic surgery is also much greater in patients whose spleen has been removed (p = 0.01), the susceptibility to microbial infection and osteomyelitis (which preferentially affects injured bone—see Additional file 2: Fig. S1B) is likely to be greater. Overall, the findings reinforce the need for vigilant long-term monitoring of bone health in patients with Gaucher disease who have undergone splenectomy.

Splenectomy has other strong associations with clinical behaviour in this condition: there was a marked association with liver disease [61], with or without pulmonary disease, as has been previously reported [62]. Indeed, most of the patients with chronic liver disease, including manifest cirrhosis, who had interstitial lung disease or pulmonary hypertension, had had their spleen removed. An unexpected finding was the greater frequency of non-haematological malignancies in patients after removal of the spleen: nine of 62, compared with 10 of 188 in the spleen-intact group (p < 0.03), Table 5. These differences are not explained by age distribution but confirm in our population, findings reported in other relatively small patient groups [63–66]. The possible effect of splenectomy on the development of various cancers in Gaucher disease has been recently reviewed [65] but the mechanism remains unclear [67]. We contend that these findings merit further exploration, particularly given the role of pathological sphingolipids in cell death, immune recognition and proliferation. That all four patients with haematological cancers were among the non-splenectomised group in our cohort, is a tantalizing matter of record.

Two hundred and forty-three patients were receiving a specific molecular therapy for Gaucher disease at the time of recruitment; in most cases enzyme therapy (velaglucerase alfa or imiglucerase) was used, but eleven patients were treated with substrate reduction therapy (eliglustat or miglustat). Three had combined therapies (eliglustat and imiglucerase; miglustat and velaglucerase alfa). At the time of writing, 65 patients, more than one quarter of the adult patients with type 1 Gaucher disease, are treated with the orally active, systemic substrate-reducing agent, eliglustat.

Specific treatment for Gaucher disease can reverse bone marrow infiltration, cytopenia and visceromegaly and ameliorate symptoms with improved quality of life. However, persistent skeletal pain is a notorious feature and an important cause of disability [48, 49, 68–70]. In this cohort, despite the emergence of molecular therapies, bone pain was present in more than half of the patients at enrolment, indicating that many UK patients had skeletal disease established before the interventions became generally available [49, 52]. Many also—especially women—had persistent fragility fractures, osteonecrosis and lytic lesions requiring orthopaedic surgery. Nonetheless, it is clear that the introduction of specific treatments exerted a protective effect on the disabling and painful consequences of osteonecrosis. In this study, disease-modifying therapy for Gaucher disease had no effect on fracture rate: this might be explained by the arrested and thus premature attainment of peak bone mass which cannot be further enhanced. As a result, the risk of fracture would be more resistant to modification. We specifically explored the effect of the age at which treatment started on either osteonecrosis or fracture, in the era of specific therapy but no association was found in this cohort. This might have resulted from the interaction of two opposing effects: once diagnosed in children and young adults, when the disease tends to be severe, early treatment is available to arrest its progress; while in older adults the condition can be severe, the later the age at which treatment is instituted may also imply intrinsically milder disease. In general, however, the interval between *diagnosis* and starting specific treatment for Gaucher disease is confirmed to be an important determinant of therapeutic outcome in the skeleton [49, 52]. The unequivocal preventative effects of enzymatic augmentation and other interventions  on osteonecrosis events, make it clear that the course of this disease can now be favourably modified.

Formerly, the diagnosis of patients with type 1 disease was mainly in adult life and often long after the onset of symptoms; those designated as type 3 Gaucher disease were nearly all identified in childhood. While this distinction reflects the often aggressive presentation of Gaucher type 3 disease, with greater awareness and improved services in the molecular era, the  trend has not continued. Ostensibly non-neuronopathic disease is increasingly recognised in children and, as we show, more subtle neuronopathic forms of the disease may develop in adults. Careful follow-up of Gaucher patients in Japan who were homozygous for the L444P *GBA1* allele but with non-neuronopathic disease in childhood, demonstrated the onset and evolution of neuronopathic features over time [71]. In the European and North-American literature [68–70, 72], as here, most patients are considered to have ‘non-neuronopathic’ rather than neuronopathic disease, despite the additional long-term risk of developing Parkinson disease and/or Lewy body dementia with accompanying neuropathology [18].

We uncovered more patients with features compatible with neuronopathic disease than expected in the UK population; these patients appear to be at a greater risk of developing early skeletal as well as other systemic manifestations of Gaucher disease [13, 73, 74]. Twenty-seven of the 250 were noted originally to have neuronopathic disease but a further 16 had features indicative of type 3 disease based principally on proven ophthalmic signs, suggesting an evolving pattern in some patients. The findings point to greater phenotypic variation than generally reported in the literature and further question the wisdom of assigning a subtype and phenotypic classification to a patient with Gaucher disease without neurological review—especially within the early years of diagnosis. With this in mind and prompted by the initial findings in a limited sample group, we plan systematically to re-evaluate the neurological features of the entire cohort. The need for intensified clinical monitoring mandated by the high risk of skeletal and other systemic manifestations in patients with neuronopathic disease will allow timely disease-modifying therapy to be introduced [33, 43]. It is now clear that diverse neurological features are found in all categories of Gaucher disease and represent a burden of illness globally which has not been fully appreciated in the Western and anglophone literature [2, 8].

The nature of this cohort, in which most patients were receiving specific treatment at the time of enrolment, required use of an analytical method that enables therapeutic stratification based on repeated evaluation of the outcomes and complications of the disease over time [44, 60]. Despite the great rarity of Gaucher disease, many clinical publications have depended on data in registries supported by the biopharmaceutical industry, which naturally place emphasis on particular products and help to meet the requirements imposed by regulatory agencies. We reasoned that an established but more independent National cohort would be needed to stratify clinical outcomes and disease behaviour in a period of intense commercial therapeutic development and would foster further research. We suggest that the study differs importantly from the international commercial registries and contend that the GAUCHERITE cohort has advantages which, *in toto,* also differentiate it from the pioneering and instructive non-commercial initiatives of investigators in individual countries such as the Netherlands, Spain and France [69, 70, 72, 73]: (1) the study represents a long-established national disease population in patients attending specialist centres at state-funded University hospitals which, unlike private healthcare, are obliged to follow agreed clinical guidelines and uniform practices; (2) all approved treatments and clinical services are funded by the UK National Health Service; (3) clinical investigators are unbiased with respect to the manufacturer of molecular therapies – all disease-related drugs and nutritional supplements and their doses are captured; (4) the study and clinical research programme is subject to a single overarching national ethical review and approval process; (5) high-level data completion across the cohort without incentives such as third-party remuneration to investigators; (6) the study requires detailed completion of structured protocols for clinical history and physical findings with serial patient-reported outcomes, including formal QOL questionnaires; (7) a single database, with mass storage of > 2500 variables, holds all radiological images of radiology and DXA scanning including linked narrative reports downloaded from the study centres; (8) use of the data is  governed by an independently approved Data Management policy, allowing investigators access to anonymised data and curated serial Biobanked blood and tissue samples linked to the relational GAUCHERITE database; (9) the research and publication policy is governed by an independent management oversight committee chaired by a senior patient advocate and charity founder.

Scrutiny of this large dataset emphasises the value of systematic phenotyping by investigators with long-term specialist engagement in the care of patients affected by an ultra-rare disease. The strength of the cohort is that it spans the pre-treatment era and subsequent interventions. The extent and depth of data is founded on repeated year-on-year serial tests (median 26) and utilises comprehensive records in a relational database that allow linear analysis using the repeated mixed measures model of individual timed events. The transformative effects of the therapies combined with intense phenotyping, time-to-event analysis and historical depth in diverse domains of the disease are also advantages.

## Conclusions

The GAUCHERITE cohort, which comprises 85% of all patients known to have Gaucher disease in the United Kingdom, represents an independent, data-rich platform to study the epidemiology and course of this heterogeneous disorder. We show that the database is a source of information that allows clinical effectiveness and appropriateness of care to be investigated in real-world clinical practice; the data can moreover be explored in depth by stratifying patients according to potentially divergent treatment effects. Finally, the cohort is an opportune resource by which to appraise newly introduced and future therapies.

## Supplementary Information

**Additional file 1.** Deep Phenotyping of Gaucher disease with supplementary materials, methods, results and references.

**Additional file 2: Fig. 1.** Magnetic resonance imaging of skeletal manifestations in Gaucher disease.

## Data Availability

The data that support the findings of this study are held by the University of Cambridge under an MRC approved Data Management Policy. Restrictions apply and data are not publicly available: applications from bona fide organizations will be considered by the University of Cambridge and clinical governance of Cambridge University NHS Foundation Trust Hospitals and also subject to approval by the Gaucherite Consortium Management Committee.

## Abbreviations

BMD: Bone mineral density
DXA: Dual-energy X-ray absorptiometry
ERT: Enzyme replacement therapy
GAUCHERITE: Gaucher Investigative Therapy Evaluation
GD-DS3: Gaucher Disease Type 1 Severity Scoring System
NHS: National Health Service
SD: Standard deviation
SRT: Substrate reduction therapy
UK: United Kingdom
