# Supplementary material for: In-depth phenotyping for clinical stratification of Gaucher disease
Source: Orphanet J Rare Dis. 2021 Oct 14;16:431. doi: 10.1186/s13023-021-02034-6 (PMC8515714; doi:10.1186/s13023-021-02034-6)
Supplement: Supplementary file 1 — Additional file 1. Deep Phenotyping of Gaucher disease with supplementary materials, methods, results and references. [file 13023_2021_2034_MOESM1_ESM.pdf]

SUPPLEMENTARY INFORMATION

MATERIALS AND METHODS (Supplementary)

Ethical considerations and Patients

With the committed interest of the patient charity, the UK Gaucher's Association, a near-complete enrolment of extant patients diagnosed with Gaucher disease in England (estimated 286 patients) was achieved. The engagement of the participants was voluntary and unpaid; study subjects were invited either by letter or by discussion at routine clinical visits. To reduce the burden placed on participants attending clinical appointments, after screening for eligibility, investigators obtained written consent – and where appropriate, assent was obtained from parents or guardians of potential participants aged 16 years or younger. Depending on their programme of clinical management, study participants who had enrolled were followed either 6 monthly, yearly or, in rare cases, biennially. No expenses, financial or otherwise, and no other material compensation or inducement was offered, or paid to subjects or their families and carers for participation. All data have been handled in conformity with international ethical, scientific and practical standards of Good Clinical Practice. The study also complies with Good Research for Comparative Effectiveness principles and the principles of the International Conference on Harmonization (1;2). The clinical project manager (KP) undertook day-to-day responsibility to monitor the clinical research activity and conduct of the study at all eight participating specialist centres.

In an effort to document the effects of molecular therapy on Gaucher disease morbidity in the context of its natural course, ethical approval has been obtained for investigators to include data obtained from the records of >50 dead patients in the UK who formerly attended

one or more of the 8 specialist centres. Some of these patients would have been naïve to enzyme replacement therapy or received the treatment late in the course of their illness.

GAUCHERITE database

The GAUCHERITE database is a bespoke database designed for the purpose of this study. The database is stored on a secure server at the principal study site. Front-end data entry is web-based for ease of access. The back-end data storage is based on Structured Query Language (SQL). SQL is a domain-specific language ideally suited for relational databases and its common usage reduces future redundancy.

The database uses Systematised Nomenclature of Medicine Clinical Terminology (SNOMED-CT), the preferred terminology recommended by NHS digital (previously known as the Health and Social Care Information Centre; HSIC). GAUCHERITE is therefore immediately compatible with NHS electronic patient records. Raw data from GAUCHERITE can be adopted into other SNOMED-CT based databases and can be mapped, using currently available tools onto databases using different terminology(s). All terms used in GAUCHERITE are clearly defined and those of special interest, for example 'avascular necrosis of bone (disorder), SCTID: 397758007' receives additional clarification to minimise ambiguity. All terms will be published on-line in a data dictionary.

Complex phenotypic terms (descriptions) will be coded using post-coordinated ontology; this confers flexibility to the database allowing relational terms to be added as required with minimal alteration (more, greater, better, increased). This is particularly useful for retrospective data entry where descriptive texts can be entered without resorting to long lists of pre-coordinated terms.

Prospective and retrospective data are collected. For retrospective data entry, the purpose is not to “digitise” historic clinical notes but to extract relevant information as recorded. Therefore, when entered, missing retrospective data are not explicitly recorded as missing.

The structure of the database has been designed for facile export of data formats as spreadsheets or comma-separated files. The exported data are readable by investigators since each variable carries two mandatory metadata; the subject identifier and date-time.

Principal Investigators and a nominated person from each specialist centre have access to the data held on the relevant study subjects. To minimise inconsistencies in reporting, retrospective data entry was restricted to the research team (database manager, data entry clerk, clinical fellows and clinical research nurse); by the same token, prospective data acquired during the conduct of the study programme was principally captured by this team.

Variables

Demographic data were collected at consent (Supplementary Table 4), which included age at time of consent, gender and educational level. Height, weight, blood pressure, temperature, pulse, assigned Gaucher disease subtype, *GBA1* genotype, treatment regimen (detailed posology of medications of special interest: enzyme therapy, nature, dose and duration; or substrate reduction therapy, bisphosphonates, calcium, vitamin D, teriparatide, denosumab, strontium ranelate) and medical history (splenectomy and cholecystectomy status, malignancies, major surgery, major bleeding, pulmonary disease, Parkinson disease, menarche, pregnancies, menopause and epilepsy). This information was reviewed and revised where necessary at every clinical appointment.

Development of the GAUCHERITE database incorporated variables from previous registries [i.e. International Collaborative Gaucher Group (ICGG) Gaucher Registry (3), International Myeloma Working Group (IMWG) (4), Gaucher Outcomes Survey (GOS) (5), Clinical Bone registry (Dendrite Database) (6) and Therapeutic goals in the treatment of Gaucher disease (7)]. To facilitate development of novel disease severity scoring systems, from the outset it was determined that every effort should be made to include a comprehensive clinical data set: more than two thousand five hundred variables are collected. Of note however, the database has been designed to capture the most commonly used severity scoring systems such as the Zimran Severity Score Index (SSI)(8), (conceived in the pre-treatment era), as well as the most recent Gaucher Disease Type 1 Severity Scoring System GD-DS3 (9). In particular, this latter Scoring System DS3, includes symptoms and manifestations of skeletal involvement associated with Gaucher disease such as the presence/absence of bone pain, together with its objective and subjective characteristics, and the presence/absence of bone crises. The presence/absence of fragility fractures, clinically defined as a fracture (including vertebral fracture) “that occurred as a result of a minimal trauma, such as a fall from standing height or less, or no identifiable trauma” according to the definition provided by the World Health Organization (10) was also recorded. Plain radiologic skeletal survey and/or magnetic resonance imaging (MRI) were used to assess the presence and approximate onset of osteonecrosis (avascular necrosis) and to document modelling deformities (i.e. Erlenmeyer flask deformity, a radiologic feature of Gaucher disease demonstrating relative constriction of the diaphysis and flaring of the metaphysis, which is usually detectable in childhood), fractures, lytic lesions, joint deformities (i.e. osteoarthritis) and replacement joint status. In addition, the patients’ self-reported incidents were captured and checked against imaging for bone-marrow infiltration, osteonecrosis, lytic lesions and determinations of bone mineral

density. Bone mineral density was assessed by dual-energy x-ray absorptiometry (DXA) at the lumbar vertebrae, hip and wrist. Values are reported as bone mineral density (BMD) g/cm^2, Z-scores and T-scores, in relation to the standard deviation from the median age and gender-specific and young adult reference values, respectively. The patient's physician reported haematological parameters (i.e. cytopaenia, bleeding), liver volume, spleen volume and status, and central nervous system, liver and lung involvement at each visit. These disease-scoring systems provide clinicians with a useful tool for long-term assessment and monitoring of patients with Gaucher disease. While they are subject to the immediate clinical judgment of the treating physician, subjective variation in the evaluation will be confounded effectively by the long-term participation and interchange of colleagues engaged in contemporary clinical practice. At the same time, specialised investigators in the UK National Health Service directed to the expert care of patients with chronic diseases will be presented with unrivalled opportunities for comparing individual real-world outcomes between individuals suffering from a chronic but heterogeneous condition, such as Gaucher disease.

Questionnaires

Questionnaires were only prospectively administered at each scheduled clinical appointment as part of GAUCHERITE (Supplementary Table 5).

Quality of life questionnaires were used as a standardised measure of health status. EuroQol 5D 5L (EQ-5D-5L) and Health Survey (SF-36) v2 EuroQol 5D 5L (EQ-5D-5L), Health Survey (SF-36) v2 (11), Quality of Life Inventory Multidimensional Fatigue Scale (PedsQL MFS) (12), Hospital Anxiety and Depression Scale (HADs) (13;14) for all adult patients (11). Additional questionnaires, including Frontal Assessment Battery (FAB) (15), The Addenbrooke's Cognitive Examination- Revised (ACE-R) (16) and Movement Disorder Society - Unified

Parkinson's Disease Rating Scale (MDS-UPDRS02) (17), were administered in patients with Parkinson disease; while FAB, ACE-R and modified Severity Screening Tool (mSST) (18) were administered in patients with type 3 (chronic neuronopathic) Gaucher disease.

Paediatric Quality of Life (PedsQL) questionnaires were used for all patients seen in paediatric specialist centres. The parents or guardians of paediatric patients were asked to complete age-specific quality of life questionnaires annually. Patients were assisted by a member of the research team to complete these in line with the guidance provided with the questionnaires. Young patients attending paediatric service continued to complete these after they undertook transitional care and moved to an adult centre. A further questionnaire was also included in paediatric patients: a paediatric development assessment tool (Vineland II) (19).

Cognitive assessment

Bedside cognitive assessments were undertaken in patients with neuronopathic Gaucher disease and patients with Gaucher-related Parkinson disease. These cognitive assessments formed part of two key objectives: to identify the presence or absence of impairment in patients and to compare the type of impairment (if identified) with any impairment found in patients with Parkinson disease associated with Gaucher disease (biallelic mutations in *GBA1*). Evidence suggests that many patients with Parkinson disease experience some mild cognitive impairment (approx. 26% (20)) which would not be identified on a standard Mini Mental State Examination (MMSE). The ACE-R incorporates both an MMSE score with a more extensive testing battery resulting in five sub-domain scores: orientation/attention, memory, verbal fluency, language and visuospatial function with greater sensitivity in identifying mild impairment (21).

The key limitation of the ACE-R is the reduction in sensitivity seen in patients who have a higher intelligence quotient or greater number of years of completed education (16). In view of this confounder to the test, the National Adult Reading Test, a short test which indicates pre-morbid intellectual function (22) was also administered on the first occasion that comprehensive cognitive testing.

The FAB was employed for all patients with neuronopathic and Gaucher-Parkinson Disease to further define the type of cognitive impairment encountered (15).

The MDS-UPDRS is the most widely used questionnaire for Parkinson disease (17). This questionnaire was used on all Gaucher patients who had a diagnosis of Parkinson disease. The mSST was used as a tool to monitor the progression of neurological manifestations in patients with neuronopathic (type 3) disease (18;23).

Imaging studies

Retrospective and prospective imaging studies were collected during the study: a total of 2398 MRI scans, 1159 plain radiographs, 933 DXA scans, 351 ultrasound studies, and 128 computerized axial tomography (CT) scans were retrieved using the Sectra Image Exchange Portal (IEP), downloaded as DICOM file formats, anonymised using a ClearCanvas workstation and archived in a Cambridge-based secure storage provided by the University of Cambridge.

SUPPLEMENTARY RESULTS

Growth, puberty, and menopause

According to self-reported growth measures reported by 223 patients over the age of 21 years, 65 patients recalled a failure to thrive in childhood, while 86 and 71 felt shorter or

underweight compared with their peers at school. Fifty-four patients recalled a delay in puberty. The median age at regular shaving in males and onset of menarche in females were 16 years (range: 9-26) and 14 years (range: 6-19), respectively. A total of 51 women were in menopause at the time of recruitment (median age 47 years, range: 26-58), and 20 patients had received or were still receiving hormone replacement treatment (HRT) at the time of recruitment (Table 1).

Gallstone disease

Seventy-nine patients had gallstone disease (median age 40 years, range: 11-66). Of these, 32 were symptomatic and prompted investigations while in 43 patients gallstones were an incidental finding (Table 4). Thirty-seven patients had a history of cholecystectomy (median age 37 years, range: 16-69 years).

REFERENCES

1. ICH Topic E 6 (R1) Guideline for Good Clinical Practice GUIDELINE FOR GOOD CLINICAL PRACTICE . Web [serial online] Available at: http://www.emea.eu.int.
2. Pieterse H, Diamant Z. Good clinical practice in clinical interventional studies. Eur Clin Respir J. 2014;1.
3. Charrow J, Andersson HC, Kaplan P et al. The Gaucher registry: demographics and disease characteristics of 1698 patients with Gaucher disease. Arch Intern Med. 2000;160:2835-2843.
4. Kyle RA, Durie BG, Rajkumar SV et al. Monoclonal gammopathy of undetermined significance (MGUS) and smoldering (asymptomatic) multiple myeloma: IMWG consensus perspectives risk factors for progression and guidelines for monitoring and management. Leukemia. 2010;24:1121-1127.
5. Zimran A, Belmatoug N, Bembi B et al. Demographics and patient characteristics of 1209 patients with Gaucher disease: Descriptive analysis from the Gaucher Outcome Survey (GOS). Am J Hematol. 2018;93:205-212.

6. Deegan PB, Pavlova E, Tindall J et al. Osseous manifestations of adult Gaucher disease in the era of enzyme replacement therapy. Medicine (Baltimore). 2011;90:52-60.

7. Pastores GM, Weinreb NJ, Aerts H et al. Therapeutic goals in the treatment of Gaucher disease. Semin Hematol. 2004;41:4-14.

8. Zimran A, Kay A, Gelbart T et al. Gaucher disease. Clinical, laboratory, radiologic, and genetic features of 53 patients. Medicine (Baltimore). 1992;71:337-353.

9. Weinreb NJ, Cappellini MD, Cox TM et al. A validated disease severity scoring system for adults with type 1 Gaucher disease. Genet Med. 2010;12:44-51.

10. Bonjour JP, Ammann P, Rizzoli R. Importance of preclinical studies in the development of drugs for treatment of osteoporosis: a review related to the 1998 WHO guidelines. Osteoporos Int. 1999;9:379-393.

11. Oemar M, Janssen B. EQ-5D-5L User Guide Basic information on how to use the EQ-5D-5L instrument. Web [serial online] Available at: https://www.unmc.edu/centric/_documents/EQ-5D-5L.pdf .

12. Varni JW, Limbers CA, Bryant WP, Wilson DP. The PedsQL multidimensional fatigue scale in pediatric obesity: feasibility, reliability and validity. Int J Pediatr Obes. 2010;5:34-42.

13. Bjelland I, Dahl AA, Haug TT, Neckelmann D. The validity of the Hospital Anxiety and Depression Scale. An updated literature review. J Psychosom Res. 2002;52:69-77.

14. Zigmond AS, Snaith RP. The hospital anxiety and depression scale. Acta Psychiatr Scand. 1983;67:361-370.

15. Kaszas B, Kovacs N, Balas I et al. Sensitivity and specificity of Addenbrooke's Cognitive Examination, Mattis Dementia Rating Scale, Frontal Assessment Battery and Mini Mental State Examination for diagnosing dementia in Parkinson's disease. Parkinsonism Relat Disord. 2012;18:553-556.

16. Komadina NC, Terpening Z, Huang Y et al. Utility and limitations of Addenbrooke's Cognitive Examination-Revised for detecting mild cognitive impairment in Parkinson's disease. Dement Geriatr Cogn Disord. 2011;31:349-357.

17. Goetz CG, Tilley BC, Shaftman SR et al. Movement Disorder Society-sponsored revision of the Unified Parkinson's Disease Rating Scale (MDS-UPDRS): scale presentation and clinimetric testing results. Mov Disord. 2008;23:2129-2170.

18. Davies EH, Surtees R, DeVile C, Schoon I, Vellodi A. A severity scoring tool to assess the neurological features of neuronopathic Gaucher disease. J Inherit Metab Dis. 2007;30:768-782.

19. Sparrow SSCDV&BDA 2005 Vineland-II Adaptive Behavior Scales:

Survey Forms Manual. Circle Pines, MN: AGS Publishing.

20. Litvan I, Aarsland D, Adler CH et al. MDS Task Force on mild cognitive impairment in Parkinson's disease: critical review of PD-MCI. Mov Disord. 2011;26:1814-1824.

21. Hsieh S, Schubert S, Hoon C, Mioshi E, Hodges JR. Validation of the Addenbrooke's Cognitive Examination III in frontotemporal dementia and Alzheimer's disease. Dement Geriatr Cogn Disord. 2013;36:242-250.

22. Crawford JR, Deary IJ, Starr J, Whalley LJ. The NART as an index of prior intellectual functioning: a retrospective validity study covering a 66-year interval. Psychol Med. 2001;31:451-458.

23. Davies EH, Mengel E, Tylki-Szymanska A et al. Four-year follow-up of chronic neuronopathic Gaucher disease in Europeans using a modified severity scoring tool. J Inherit Metab Dis. 2011;34:1053-1059.

24. Tylki-Szymańska A, Czartoryska B, Vanier MT, Poorthuis BJ, Groener JA, Ługowska A, Millat G, Vaccaro AM, Jurkiewicz E. Non-neuronopathic Gaucher disease due to saposin C deficiency. Clin Genet. 2007;72:538-542.

2

LEGENDS TO SUPPLEMENTARY FIGURE

Supplementary Figure 1. Magnetic resonance imaging of the skeleton in Gaucher disease

Hypointense T1-weighted signals due to osteonecrosis of the right femoral head, which displays secondary osteoarthritic changes (A). Hypointense signals due to osteonecrosis within the proximal left tibia (B). Bilateral bone marrow infarctions in the distal femurs and osteomyelitis in the right distal femur with involucrum and lateral sinus tract to the skin (C). Bone marrow infiltration by Gaucher cells showing a patchy low T1w marrow signal along all lumbar vertebrae (D, right panel). On STIR sequences, there is minor diffuse bright T2w signal within the central vertebral marrow (D, left panel).

SUPPLEMENTARY TABLES

Supplementary Table 1. Gaucher disease subtypes

Characteristic	Type 1 Chronic, non-neuronopathic	Type 2 Acute, neuronopathic	Type 3 Subacute, neuronopathic
**Age at onset**	Childhood/adulthood	Infancy	Childhood/adolescence
**Disease onset**	Heterogeneous	Stereotypic	Heterogeneous
**Disease course**	Progressive	Rapidly progressive	Progressive
**Hepatosplenomegaly**	Present	Present	Present
**Skeletal disease**	Present	Absent	Present
**Primary CNS disease**	Absent*	Present	Present
**Other systems**	Hepatic fibrosis<br>Pulmonary hypertension	Congenital ichthyosis	Pulmonary infiltrates<br>Kyphoscoliosis<br>(calcified aortic & mitral valves, corneal opacities,hydrocephalus‡)
**Life expectancy§**	Childhood/adulthood	Death by 3 years	Childhood/adulthood
**Ethnicity**	Panethnic/Ashkenazi Jews	Panethnic	Panethnic/Norrbottnian Sweden
**Genetics**	Autosomal recessive	Autosomal recessive	Autosomal recessive
**Mutation association†**	N370S and others	Diverse	L444P

* Late-onset Parkinsonism and Lewy body dementia occur in a minority

§ Life expectancy before the enzyme replacement therapy (ERT) era

† Exemplary homozygous genotypes

‡ Features specific to D409H homozygotes

Supplementary Table 2. Specialist centres

Hospital	N=patients recruited
Cambridge University Hospitals, Cambridge*	89
Royal Free Hospital, London*	99
National Hospital for Neurology and Neurosurgery*,	8
Salford Royal Hospital, Manchester*	24
Queen Elizabeth Hospital, Birmingham*	5
Royal Manchester Children’s Hospital, Manchester+	11
Great Ormond Street Hospital (GOSH), London+	11
Birmingham Women’s and Children’s Hospital, Birmingham+	3
**Total**	**250**

Adult/Paediatric +*

Supplementary Table 3. Data collection

**Variable category**
Demographics
Treatment Regime
Medical History
Imaging: magnetic resonance imaging, dual energy X-Ray absorptiometry, computed tomography, ultrasound, X-Ray
Pathology: liver, renal, bone, lipid, platelet count, haemoglobin concentration, white cell enzyme, PARC/CCL18, chitotriosidase, fibroblast enzyme activity, protein electrophoresis, urine analysis (creatinine, albumin and albumin creatinine ratio
Vital signs
Questionnaires

Supplementary Table 4. Demographic variables

Domain name
Subject ID (Unique)
Secondary ID (Unique)
Gender (Male/Female)
Study Site Number
Date signed consent
Date reconsented to SubAmd 2
Did you obtain further qualifications after leaving full time education?
Are your parents biologically related (consanguineous)?
Age left full time education
Are you Jewish?
Meets inclusion criteria
Meets exclusion criteria

Supplementary Table 5. Summary of visits

	**Screening**	**Baseline**	**Follow up/Observation**							**Early Termination**
Visit	V1	V2	V3	V4	V5	V6	V7	V8	V9	ET
Month	(-1 to -2)	0	Month 6	Month 12	Month 18	Month 24	Month 30	Month 36	Month 42	ET
Visit Window [(a)]			±3 Months	±3 Months	±3 Months	±3 Months	±3 Months	±3 Months	±3 Months	
Informed Consent	X									
Demographics	X									
Medical History[(b)]	X	X	X	X						
Review Entry Criteria	X	X								
Review Gaucher Medications	X	X	X	X	X	X	X	X	X	X
Medications Review	X	X	X	X	X	X	X	X	X	X
Height	X	X	X	X	X	X	X	X	X	X
Weight	X	X	X	X	X	X	X	X	X	X
Vital Signs	X	X	X	X	X	X	X	X	X	X

Physical examination	X	X	X	X	X	X	X	X	X	X
Blood sample for genetic testing [(c)]	X	X	X	X	X	X	X	X	X	X
Blood Collection for routine tests	X	X	X	X	X	X	X	X	X	X
Blood Collection for research tests [(e)]	X	X	X	X	X	X	X	X	X	X
Urine collection for research	X	X	X	X	X	X	X	X	X	X
Cerebrospinal fluid (CSF)[(d)] collection	X	X	X	X	X	X	X	X	X	X
Review of Clinical Events		X	X	X	X	X	X	X	X	X
Health Status Questionnaires	X	X	X	X	X	X	X	X	X	X
Neurological Questionnaires	X	X	X	X	X	X	X	X	X	X
Review Drug History	X	X								
Brain MRI[(d)]	X	X	X	X	X	X	X	X	X	X
Brain DTI[(d)]	X	X	X	X	X	X	X	X	X	X

Skeletal MRI[(d)]	X	X	X	X	X	X	X	X	X	X
Bone densitometry[(d)]	X	X	X	X	X	X	X	X	X	X
Magnetic Resonance Spectroscopy (MRS) [(d)]	X	X	X	X	X	X	X	X	X	X
Bone Marrow Sample [(d)]	X	X	X	X	X	X	X	X	X	X
UPSIT and/or Sniffin' Stick [(f)]	X	X	X	X	X	X	X	X	X	X
Skin Biopsy (g)	X	X	X	X	X	X	X	X	X	X

(a) Data collected clinically associated to the most appropriate visit

(b) Medical History obtained at visits after screening to ensure all patient data are captured

(c) Only one blood sample for genetic testing is taken during the study

(d) Only if part of routine clinical management

(e) At each clinical visit when possible

(f) Carried out if available

(g) Only one skin biopsy obtained during the study for research purposes if facilities are available and consent obtained.

Supplementary Table 6. Splenectomy status and medications of the GAUCHERITE cohort and subgroups at recruitment

Variables	Categories*	Entire cohort (N=250)	Paediatric (N=26)	Adult (N=224)	Gaucher Type 1 (N=223)	Gaucher Type 3 (N=27)
Splenectomy status	Yes	62 (25)	-	62 (28)	53 (24)	9 (33)
	Age at splenectomy, years, mean ± SD	20±14	-	20±14	22±14	5±4
	Median (range)	17 (1-58)	-	17 (1-58)	21 (2-58)	5 (1-15)
	No	188 (75)	26 (100)	162 (72)	170 (76)	18 (67)
ERT/SRT treatment status at +/- 6 months of recruitment	N.	243 (97)	26 (100)	219 (98)	218 (98)	25 (93)
	Alglucerase	-	-	-	-	-
	Eliglustat	7 (3)	1 (4)	6 (3)	6 (3)	1 (4)
	Imiglucerase	89 (37)	2 (7)	87 (40)	79 (36)	9 (36)
	Miglustat	4 (2)	-	4 (2)	4 (2)	-
	Taliglucerase	-	-	-	-	-
	Velaglucerase alfa	141 (58)	22 (85)	119 (54)	128 (59)	14 (56)
	Combined ERT & SRT[#,§]	2 (1)	1 (4)[§]	1 (1)[#]	1 (<1)[§]	1 (4)[#]
Bone treatment status at +/- 6 months of recruitment	N.	94 (38)	2 (8)	92 (41)	86 (39)	8 (30)
	Calcium/vitamin D supplements	79 (84)	2 (100)	77 (84)	72 (84)	7 (87.5)
	Anti-resorptive drugs	8 (9)	-	8 (9)	8 (9)	-
	Anti-resorptive drugs & calcium/vitamin D	6 (6)	-	6 (6)	6 (7)	-
	Anabolic drugs & calcium/vitamin D	1 (1)	-	1 (1)	-	1 (12.5)

*Categorical variables are expressed as frequency (percent). Continuous variables are expressed as mean ± SD and median (range).

Abbreviations: ERT, enzyme replacement treatment; SD, standard deviation; SRT, substrate reduction therapy.

[#]Combined eliglustat and imiglucerase; [§]Combined miglustat and velaglucerase alfa.

Supplementary Table 7. *GBA1* Genotypes and Phenotype Classification of Study Cohort

Nº	Subtype	Allele 1	Mutation 1	Allele 2	Mutation 2
1	1	Y22C	c.182A>G	F259L	c.894C>A
1†	1	not done†	not done†	not done†	not done†
2	1	L444P	c.1448T>C	R496H	c.1604G>A
3	1	c.589-2A>G	c.589-2A>G	N370S	c.1226A>G
4	1	N370S	c.1226A>G	L444P+ E326K	c.1448T>C
5	1	N370S	c.1226A>G	L444P	c.1448T>C
6	1	RecNcil	c.1488T>C/c.1483G>C/ c.1497G>C	R463C	c.1504C>T
7	1	N370S	c.1226A>G	RecNcil	c.1488T>C/c.1483G>C/ c.1497G>C
8	1	N370S	c.1226A>G	R463C	c.1504C>T
9	1	N370S	c.1226A>G	R463C	c.1504C>T
10	1	203dupC	c.203dupC	R463C	c.1504C>T
11	3	L444P	c.1448T>C	D409H	c.1342G>C
12	1	N370S	c.1226A>G	L444P	c.1448T>C
13	1	N370S	c.1226A>G	N370S	c.1226A>G
14	1	c.184T>G	c.184T>G	R496H	c.1604G>A
15	1‡	RecNcil	c.1488T>C/c.1483G>C/ c.1497G>C	R463C	c.1504C>T
16	1	A318D	c.1070C>A	N370S	c.1226A>G
17	1	N370S	c.1226A>G	D218V	c.770A>T
18	1	N370S	c.1226A>G	R120W	c.475C>T
19	1	N370S	c.1226A>G	RecNcil	c.1488T>C/c.1483G>C/ c.1497G>C
20	1	P182T	c.661C>A	N370S	c.1226A>G
21	1	N370S	c.1226A>G	N370S	c.1226A>G
22	3	R463C	c.1504C>T	D409H	c.1342G>C
23	1‡	L444P	c.1448T>C	R463C	c.1504C>T
24	1	N370S	c.1226A>G	D474N	c.1537G>A
25	1	R120W	c.475C>T	N370S	c.1226A>G
26	1	N370S	c.1226A>G	N370S	c.1226A>G
27	1	N370S	c.1226A>G	RecNcil + RecΔ5 + T369M	c.1488T>C/c.1483G>C/ c.1497G>C; c.1263del55 c.1223C>T
28	1	L444P	c.1448T>C	R463C	c.1504C>T
29	1	N370S	c.1226A>G	N370S	c.1226A>G
30	1	N370S	c.1226A>G	N370S	c.1226A>G
31	1	N370S	c.1226A>G	N370S	c.1226A>G
32	1	N370S	c.1226A>G	Y418S	c.1370A>C
33	1	N370S	c.1226A>G	R257Q	c.887G>A
34	1	N370S	c.1226A>G	Complex C (Rec5a)	c.667T>C/c.754T>A/ c.703T>C/ c.721G>A/c.475C>T/ c.681T>G/c.689T>G
35	1	Y22C	c.182A>G	F259L	c.894C>A
36	1	N370S	c.1226A>G	c.589_2A>G	c.589_2A>G

37	1	RecA456P	c.1448T>C/ c.1483G>C	N188S	c.680A>G
38	1	N370S	c.1226A>G	c.1388+1G>A	c.1388+1G>A
39	1	N370S	c.1226A>G	D315H	c.1060G>C
40	1	N370S	c.1226A>G	N370S	c.1226A>G
41	1	N370S	c.1226A>G	N370S	c.1226A>G
42	1	N370S	c.1226A>G	L444P	c.1448T>C
43	1	L105R	c.431T>G	N370S	c.1226A>G
44	1	N370S	c.1226A>G	c.334_338delCAGAA	c.334_338delCAGAA
45	1	N370S	c.1226A>G	L444P	c.1448T>C
46	1	N370S	c.1226A>G	D409H + H255Q	c.1342G>C; c.882T>G
47	1	N370S	c.1226A>G	IVS 2+1G>A	c.115+1G>A
48	1	N370S	c.1226A>G	L444P	c.1448T>C
49	1	N370S	c.1226A>G	Y220C	c.776A>G
50	1	N370S	c.1226A>G	L444P	c.1448T>C
51	1	N370S	c.1226A>G	L444P	c.1448T>C
52	3	L444P	c.1448T>C	L444P	c.1448T>C
53	1	N370S	c.1226A>G	P182T	c.661C>A
54	1	RecNcil + RecΔ5	c.1488T>C/c.1483G>C/ c.1497G>C; c.1263del55	F216Y	c.764T > A
55	1	N370S	c.1226A>G	Y418C	c.1370A>G
56	1‡	R463C	c.1504C>T	IVS 2+ 1G>A	c.115+1G>A
57	1	R463C	c.1504C>T	R463C	c.1504C>T
58	1	N370S	c.1226A>G	L444P	c.1448T>C
59	1	L444P	c.1448T>C	c.153-154insTACAGC	c.148 _ 153dupTACAGC
60	1	N370S	c.1226A>G	L480P	c.1556T>C
61	1	N370S	c.1226A>G	L444P	c.1448T>C
62	1	N370S	c.1226A>G	RecNcil	c.1488T>C/c.1483G>C/ c.1497G>C
63	1	R496H	c.1604G>A	G202R	c.721G>A
64	1	N370S	c.1226A>G	L444P	c.1448T>C
65	3	R463C	c.1504C>T	RecNcil	c.1488T>C/c.1483G>C/c.1 497G>C
66	1	N370S	c.1226A>G	L444P	c.1448T>C
67	1‡	RecNcil	c.1488T>C/c.1483G>C/ c.1497G>C	R463C	c.1504C>T
68	1	N370S	c.1226A>G	84GG	c.84dupG
69	1	N370S	c.1226A>G	R502H	c.1505C>T
70	1‡	R463C	c.1504C>T	G377R	c.1246G>C
71	1‡	P266R	c.914C>G	L444P	c.1448T>C
72	1	N370S	c.1226A>G	RecNcil	c.1488T>C/c.1483G>C/ c.1497G>C
73	1	N370S	c.1226A>G	L444P	c.1448T>C
74	1	N370S	c.1226A>G	RecNcil	c.1488T>C/c.1483G>C/ c.1497G>C
75	1	S173X	c.635C>G	N370S	c.1226A>G
76	1	N370S	c.1226A>G	c.316_316delC	c.316_316delC
77	1	N370S	c.1226A>G	c.316_316delC	c.316_316delC
78	1	N370S	c.1226A>G	S173X	c.635C>G
79	1	N370S	c.1226A>G	L383P	c.1265T>C
80	1	N370S	c.1226A>G	RecNcil	c.1488T>C/c.1483G>C/ c.1497G>C

81	1	N370S	c.1226A>G	N370S	c.1226A>G
82	1	N370S	c.1226A>G	IVS 2+1G>A + c.44T>C + c.46A>G	c.115+1G>A c.44T>C/c.46A>G/
83	1	N370S	c.1226A>G	L444P	c.1448T>C
84	3	L444P	c.1448T>C	L444P	c.1448T>C
85	3	L444P	c.1448T>C	L444P	c.1448T>C
86	1	N370S	c.1226A>G	L444P	c.1448T>C
87	1	N370S	c.1226A>G	RecNcil<br><br>+ RecΔ5	c.1488T>C/c.1483G>C/ c.1497G>C;<br>c.1263del55
88	1	N370S	c.1226A>G	L444P	c.1448T>C
89	1	N370S	c.1226A>G	RecNcil	c.1488T>C/c.1483G>C/ c.1497G>C
90	1	N370S	c.1226A>G	I402T + E236K	c.1322T>C + c.1093G>A
91	3	L444P	c.1448T>C	L444P	c.1448T>C
92	1	N370S	c.1226A>G	V394L	c.1297G>T
93	1	IVS 2+1G>A	c.115+1G>A	N370S	c.1226A>G
94	1‡	R262G	c.901C>G	RecNcil	c.1488T>C/c.1483G>C/ c.1497G>C
95	1	N370S	c.1226A>G	N370S	c.1226A>G
96	1	L444P	c.1448T>C	N370S	c.1226A>G
97	1	IVS 2+1G>A	c.115+1G>A	N370S	c.1226A>G
98	1	N370S	c.1226A>G	D409H + H255Q	c.1342G>C;c.882T>G
99	1	N370S	c.1226A>G	N370S	c.1226A>G
100	1	N370S	c.1226A>G	84GG	c.84dupG
101	1	N370S	c.1226A>G	N370S	c.1226A>G
102	1	N370S	c.1226A>G	R463C	c.1504C>T
103	1	N370S	c.1226A>G	L444P	c.1448T>C
104	1	N370S	c.1226A>G	84GG	c.84dupG
105	1	N370S	c.1226A>G	N370S	c.1226A>G
106	1	N370S	c.1226A>G	84GG	c.84dupG
107	3	L444P	c.1448T>C	L444P	c.1448T>C
108	1	N370S	c.1226A>G	L444P	c.1448T>C
109	1	N370S	c.1226A>G	N370S	c.1226A>G
110	1	N370S	c.1226A>G	N370S	c.1226A>G
111	1	N370S	c.1226A>G	L444P	c.1448T>C
112	1	N370S	c.1226A>G	L444P	c.1448T>C
113	1	N370S	c.1226A>G	N370S	c.1226A>G
114	1	R463C	c.1504C>T	595-596delCT	c.595_596delCT
115	1	N370S	c.1226A>G	D380A	c.1256A>C
116	1	N370S	c.1226A>G	84GG	c.84dupG
117	1	N370S	c.1226A>G	L444P	c.1448T>C
118	1	N370S	c.1226A>G	L444P	c.1448T>C
119	1	D409H	c.1342G>C	N370S	c.1226A>G
120	1	N370S	c.1226A>G	N370S	c.1226A>G
121	1	N370S	c.1226A>G	N370S	c.1226A>G
122	1	N370S	c.1226A>G	RecNcil	c.1488T>C/c.1483G>C/c.1 497G>C
123	1	N370S	c.1226A>G	G250V	c.866G>T
124	1	N370S	c.1226A>G	L444P	c.1448T>C
125	1	N370S	c.1226A>G	N370S	c.1226A>G
126	3	L444P	c.1448T>C	R463C	c.1504C>T
127	1	N370S	c.1226A>G	c.1388+1G>A	c.1388+1G>A

128	1	N370S	c.1226A>G	RecTL (D409H + L444P + A456P + V460V)	c.1342G>T, c.1448T>C, c.1483G>C, c.1497G>C
129	1‡	L444P	c.1448T>C	P266A	c.913C>G
130	1	N370S	c.1226A>G	V447E	c.1457T>A
131	1	N370S	c.1226A>G	D409H + H255Q	c.882T>G/c.1342G>C
132	1	N370S	c.1226A>G	D409H	c.1342G>C
133	1	N370S	c.1226A>G	84GG	c.84dupG
134	1	N370S	c.1226A>G	RecNcil + RecΔ5	c.1488T>C/c.1483G>C/ c.1497G>C; c.1263del55
135	1	N370S	c.1226A>G	N370S	c.1226A>G
136	3	L444P	c.1448T>C	L444P	c.1448T>C
137	1	N370S	c.1226A>G	RecNcil	c.1488T>C/c.1483G>C/c.1 497G>C
138	3	L444P	c.1448T>C	L444P	c.1448T>C
139	1	N370S	c.1226A>G	N370S	c.1226A>G
140	1	N370S	c.1226A>G	R257Q	c.887G > A
141	1	84GG	c.84dupG	R496H	c.1604G>A
142	1	N370S	c.1226A>G	c.1388+1G>A	c.1388+1G>A
143	1	N370S	c.1226A>G	R120Q	c.476G>A
144	1‡	RecNcil	c.1488T>C/c.1483G>C/ c.1497G>C	R463C	c.1504C>T
145	1	N370S	c.1226A>G	84GG	c.84dupG
146	1	D409H + H255Q	c.882T>G; c.1342G>C	N370S	c.1226A>G
147	1	N370S	c.1226A>G	R359X	c.1192C>T
148	1	N370S	c.1226A>G	RecNcil + L197F	c.1488T>C/c.1483G>C/ c.1497G>C + c.706C>T
149	1	N370S	c.1226A>G	L444P	c.1448T>C
150	1	N370S	c.1226A>G	84GG	c.84dupG
151	3	L444P	c.1448T>C	L444P	c.1448T>C
152	1	N370S	c.1226A>G	L444P	c.1448T>C
153	1	N370S	c.1226A>G	N370S	c.1226A>G
154	1	N370S	c.1226A>G	L444P	c.1448T>C
155	1	N370S	c.1226A>G	R120Q	c.476G>A
156	1	N370S	c.1226A>G	84GG	c.84dupG
157	1	R496H	c.1604G>A	R359X	c.1192C>T
158	1	R262G	c.901C>G	R262G + RecNcil	c.901C>G + c.1488T>C/c.1483G>C/ c.1497G>C
159	1	N370S	c.1226A>G	P387L	c.1277C>T
160	1	N370S	c.1226A>G	L444P	c.1448T>C
161	1	N370S	c.1226A>G	c.1529delC	c.1529delC
162	1	N370S	c.1226A>G	L105R	c.431T>G
163	1	N370S	c.1226A>G	N370S	c.1226A>G
164	1	L444P	c.1448T>C	R463C	c.1504C>T
165	1	W184R	c.667T>C	W184R	c.667T>C
166	1	N370S	c.1226A>G	N370S	c.1226A>G
167	1‡	L444P	c.1448T>C	R463C	c.1504C>T
168	1	L444P	c.1448T>C	F216Y	c.764T>A
169	1‡	L444P	c.1448T>C	R463C	c.1504C>T
170	3	L444P	c.1448T>C	L444P	c.1448T>C
171	1	N370S	c.1226A>G	L444P	c.1448T>C
172	1	N370S	c.1226A>G	N370S	c.1226A>G

173	1	N370S	c.1226A>G	N370S	c.1226A>G
174	3	L444P	c.1448T>C	L444P	c.1448T>C
175	1	N370S	c.1226A>G	N370S	c.1226A>G
176	1	L66P	c.314T>C	N370S	c.1226A>G
177	1	R463C	c.1504C>T	R463C	c.1504C>T
178	1	N370S	c.1226A>G	G234E	c.701G>A
179	1	N370S	c.1226A>G	N370S	c.1226A>G
180	1	N370S	c.1226A>G	L444P	c.1448T>C
181	1	N370S	c.1226A>G	N370S	c.1226A>G
182	1	N370S	c.1226A>G	L444P	c.1448T>C
183	1	D409H + H255Q	c.882T>G; c.1342G>C	N370S	c.1226A>G
184	1	N370S	c.1226A>G	L444P	c.1448T>C
185	1	N370S	c.1226A>G	R496H	c.1604G>A
186	3	L444P	c.1448T>C	L444P	c.1448T>C
187	1	N370S	c.1226A>G	N370S	c.1226A>G
188	1	N370S	c.1226A>G	N370S	c.1226A>G
189	1	T323I	c.1085C>T	N370S	c.1226A>G
190	3	L444P	c.1448T>C	L444P	c.1448T>C
191	3	L444P	c.1448T>C	L444P	c.1448T>C
192	3	L240V	c.835C>G	L240V	c.835C>G
193	1‡	R257Q	c.887G>A	R463C	c.1504C>T
194	3	E233D	c.816A>C	L444P	c.1448T>C
195	1‡	H311R	c.1049A>G	R359Q	c.1193G>A
196	3	L444P	c.1448T>C	D409H	c.1342G>C
197	1	N370S	c.1226A>G	RecΔ5	c.1263del55
198	1	N370S	c.1226A>G	RecTL (D409H + L444P + A456P + V460V)	c.1342G>T, c.1448T>C, c.1483G>C, c.1497G>C
199	1	R463C	c.1504C>T	L444P	c.1448T>C
200	3	L444P	c.1448T>C	L444P	c.1448T>C
201	1	R359Q	c.1193G>A	I132F	c.394A>T
202	1	L444P	c.1448T>C	R463C	c.1504C>T
203	1	N370S	c.1226A>G	S488F/S488L	c.1580C>T
204	3	L444P	c.1448T>C	L444P	c.1448T>C
205	1	R131C	c.508C>T	R463C	c.1504C>T
206	1	N370S	c.1226A>G	R359X	c.1192C>T
207	1	N370S	c.1226A>G	N370S	c.1226A>G
208	1	N370S	c.1226A>G	N370S	c.1226A>G
209	1	N370S	c.1226A>G	RecNcil	c.1448T>C
210	3	L444P	c.1448T>C	L444P	c.1448T>C
211	1	N370S	c.1226A>G	N462K	c.1503C>G
212	1	N370S	c.1226A>G	L444P	c.1448T>C
213	1	L444P	c.1448T>C	N370S	c.1226A>G
214	1	N370S	c.1226A>G	R120W	c.475C>T
215	3	L444P	c.1448T>C	L444P	c.1448T>C
216	1	N370S	c.1226A>G	c.1249_1251 del TGG	c.1249_1251 del TGG
217	1	N370S	c.1226A>G	R131C	c.508C>T
218	1‡	R463C	c.1504C>T	L444P	c.1448T>C
219	1	N370S	c.1226A>G	RecA456P (L444P + A456P)	c.1448T>C/c.1483G>C

220	1	N370S	c.1226A>G	RecA456P (L444P + A456P)	c.1448T>C/c.1483G>C
221	1	N370S	c.1226A>G	RecNcil	c.1488T>C/c.1483G>C/ c.1497G>C
222	1	N370S	c.1226A>G	L444P	c.1448T>C
223	3	L444P	c.1448T>C	L444P	c.1448T>C
224	1	N370S	c.1226A>G	L444P	c.1448T>C
225	1‡	R463C	c.1504C>T	R496C	c.1603C>T
226	1	R463C	c.1504C>T	N462K	c.1503C>G
227	1	L444P	c.1448T>C	R463C	c.1504C>T
228	1	L444P	c.1448T>C	R463C	c.1504C>T
229	1	N370S	c.1226A>G	L444P	c.1448T>C
230	1	N370S	c.1226A>G	R120W	c.475C>T
231	1	F397S	c.1307T>C	c.334_338delcagaa	c.334_338delcagaa
232	1	N370S	c.1226A>G	RecA456P (L444P + A456P)	c.1448T>C c.1483G>C
233	1	N370S	c.1226A>G	IVS 2+1G>A	IVS 2+1G>A
234	3	L444P	c.1448T>C	L444P	c.1448T>C
235	1	N370S	c.1226A>G	D24Y	c.187G>T
236	1	84GG	c.84dupG	N370S	c1226A>G
237	1	N370S	c.1226A>G	RecΔ5	c.1263–1317del55
238	3	L444P	c.1448T>C	L444P	c.1448T>C
239	1	N370S	c.1226A>G	RecNcil	(L444P + A456P + V460V)
240	1	N370S	c.1226A>G	RecΔ5	c.1263-1317del55
241	1	N370S	c.1226A>G	RecΔ5	c.1263-1317del55
242	1‡	RecNcil	c.1488T>C/c.1483G>C/c.1497G>C	R463C	c.1504C>T
243	1	N370S	c.1226A>G	RecA456P (L444P + A456P)	c.1448T>C c.1483G>C
244	1	N370S	c.1226A>G	L444P	c.1448T>C
245	1	P182T	c.661C>A	N370S	c.1226A>G
246	1	W184R	c.667T>C	N370S	c.1226A>G
247	1	N370S	c.1226A>G	L444P	c.1448T>C
248	1	RecNcil<br>+RecΔ5	c.1488T>C/c.1483G>C/ c.1497G>C;<br>c.1263-1317del55	N370S	c.1226A>G
249*	1	M1L	c.1 A->T*	L349P*	c.1046 T->C*

‡ Classified as Gaucher disease type 1 at baseline; neuronopathic features later identified (see main text)

**PSAP* mutations in adult with Gaucher phenotype due to SAP-C deficiency (24).
