# Supplementary figures and images for: In-depth phenotyping for clinical stratification of Gaucher disease
Source: Orphanet J Rare Dis. 2021 Oct 14;16:431. doi: 10.1186/s13023-021-02034-6 (PMC8515714; doi:10.1186/s13023-021-02034-6)

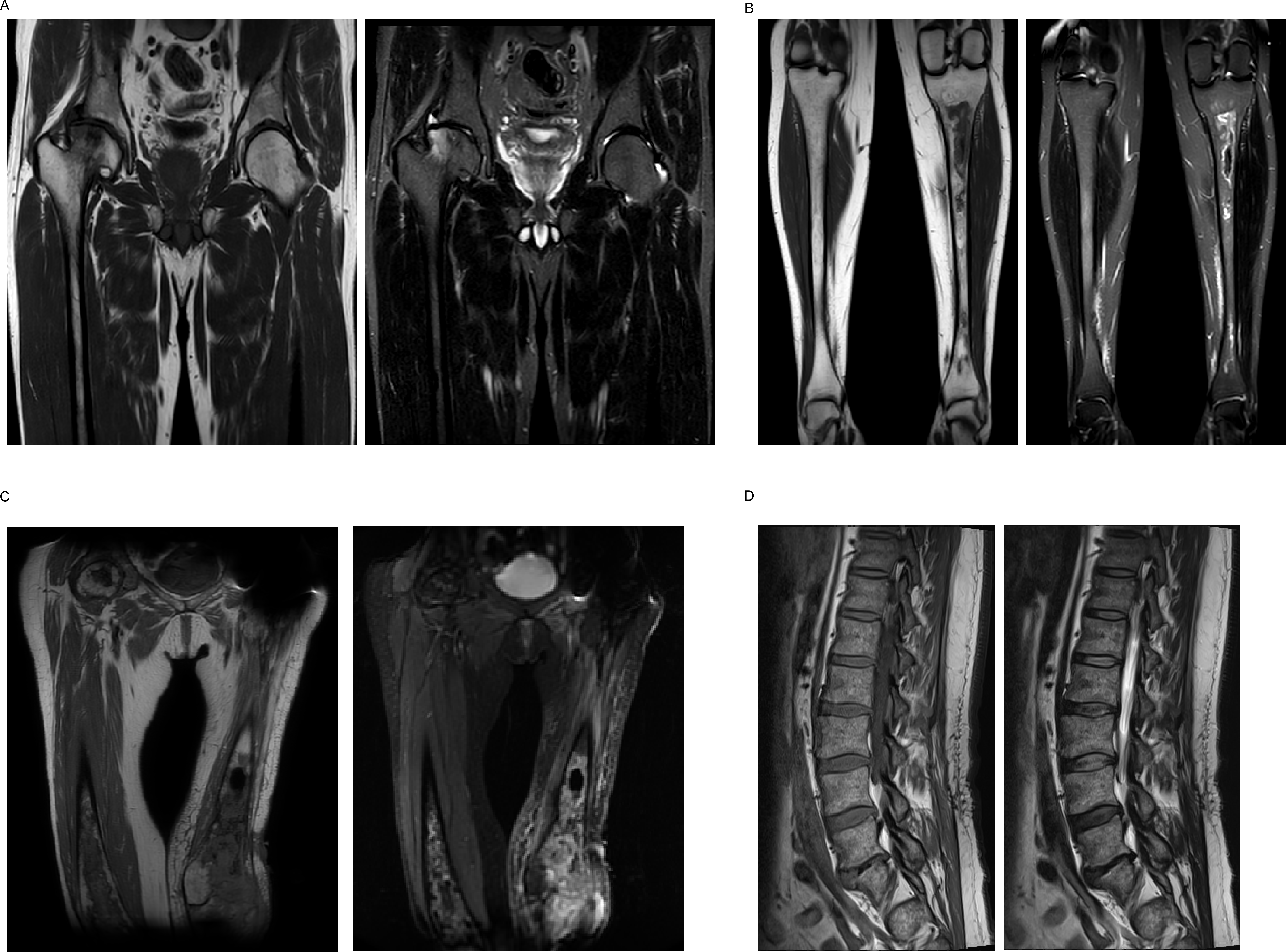

Supplement: Supplementary file 2 — Additional file 2: Fig. 1. Magnetic resonance imaging of skeletal manifestations in Gaucher disease. [file 13023_2021_2034_MOESM2_ESM.tif]
